# Cobalt-Catalyzed Cyclization of 2-Bromobenzamides with Carbodiimides: A New Route for the Synthesis of 3-(Imino)isoindolin-1-ones

**DOI:** 10.3390/molecules26237212

**Published:** 2021-11-28

**Authors:** Hasil Aman, Yu-Chiao Huang, Yu-Hao Liu, Yu-Lin Tsai, Min Kim, Jen-Chieh Hsieh, Gary Jing Chuang

**Affiliations:** 1 Department of Chemistry, Chung Yuan Christian University, Taoyuan 320314, Taiwan; hasilaman786@gmail.com (H.A.); jojohuang1234567890@gmail.com (Y.-C.H.); ytfdfvhhbbn@gmail.com (Y.-H.L.); 2 Department of Chemistry, Tamkang University, New Taipei City 251301, Taiwan; zzz759153@gmail.com; 3 Department of Chemistry, Chungbuk National University, Cheongju 28644, Korea; minkim@chungbuk.ac.kr

**Keywords:** cobalt-catalyzed, *N*-heterocycle, cyclization, carbodiimide, 3-(imino)isoindolin-1-one

## Abstract

A novel synthetic pathway to approach 3-(imino)isoindolin-1-ones by the Co-catalyzed cyclization reaction of 2-bromobenzamides with carbodiimides has been developed. This catalytic reaction can tolerate a variety of substituents and provide corresponding products in moderate yields for most cases. According to the literature, the reaction mechanism is proposed through the formation of a five-membered aza-cobalacycle complex, which carries out the following reaction subsequence, including nucleophilic addition and substitution, to furnish the desired structures.

## 1. Introduction

3-(Imino)isoindolin-1-one, an interesting *N*-heterocycle with two unsymmetrically unsaturated C-X functional groups on the five-membered indole core, has attracted attention from chemists in various fields due to its uncommon structure. However, this unique structure has also enhanced the difficulty of its synthetic pathways; thus, only limited reports have described synthetic methods to approach the derivatives of 3-(imino)isoindolin-1-one [1,2,3,4,5,6,7,8,9,10,11,12,13,14]. Among the reported methodologies, transition-metal-catalyzed cyclization reactions represent highly efficient processes that allow synthesis with fewer steps and higher yields. This progress, accompanied by higher efficient synthesis, is significantly beneficial for providing related analogs and able to satisfy the different structural requirements in various fields, which is reported to improve the potential of industrial applications.

**Scheme 1:**
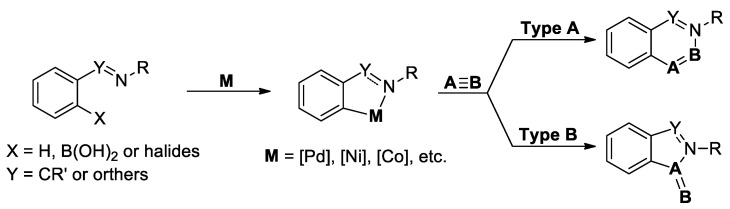
Cyclization through the five-membered metallacycle.

**Scheme 2:**
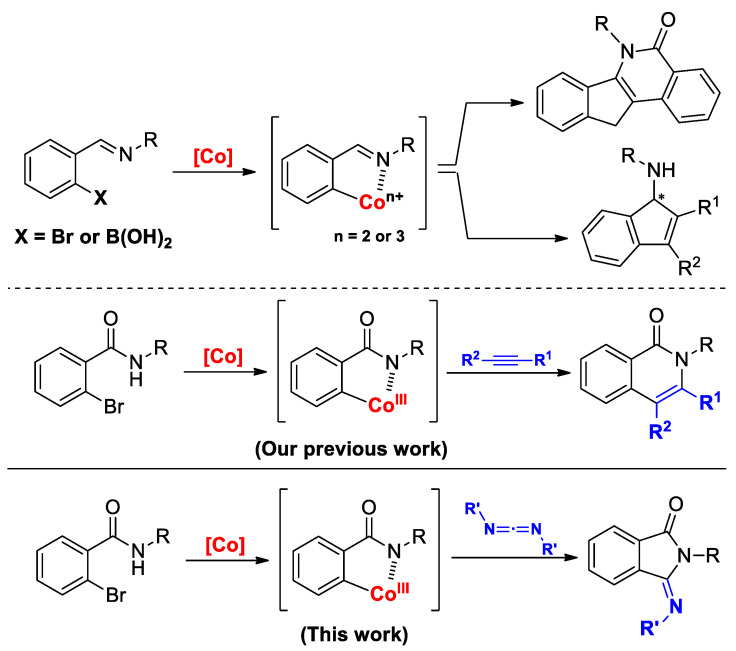
Cobalt-catalyzed cyclization reactions.

For the reported transition-metal catalytic cyclization, the most frequently adopted principle is through the formation of a five-membered metallacycle as the key intermediate. This five-membered intermediate can easily combine with subsequent steps to create diverse *N*-heterocycles (Scheme 1). A variety of late-transition-metals have been shown to possess the ability to form this intermediate for further transformations [5,7,15,16,17,18,19,20,21,22,23,24], which have been commonly indicated to construct six- or five-membered *N*-heterocyclic structures via the 1,2- or 1,1-insertion of molecules containing multiple bonds. For those revealed catalysts, the first-row transition-metal complexes exhibit comparably higher economic efficiency, and our continuing studies on cobalt-catalyzed coupling reactions [25], as well as the experiences in cyclization reactions [26,27,28,29,30,31,32,33,34,35], encouraged us to attempt the synthesis of diverse *N*-heterocyclic scaffolds by using the five-membered cobalacycle intermediate and switching different cyclization partners (Scheme 2). Under this reaction concept, we were able to obtain several *N*-heterocyclic compounds, including indenoisoquinolinones, chiral 1-aminoindenes, and isoquinolones. Further study by surveying carbodiimide as the coupling partner under a similar reaction protocol allowed us to obtain 3-(imino)isoindolin-1-ones.

## 2. Results and Discussion

**Scheme 3:**
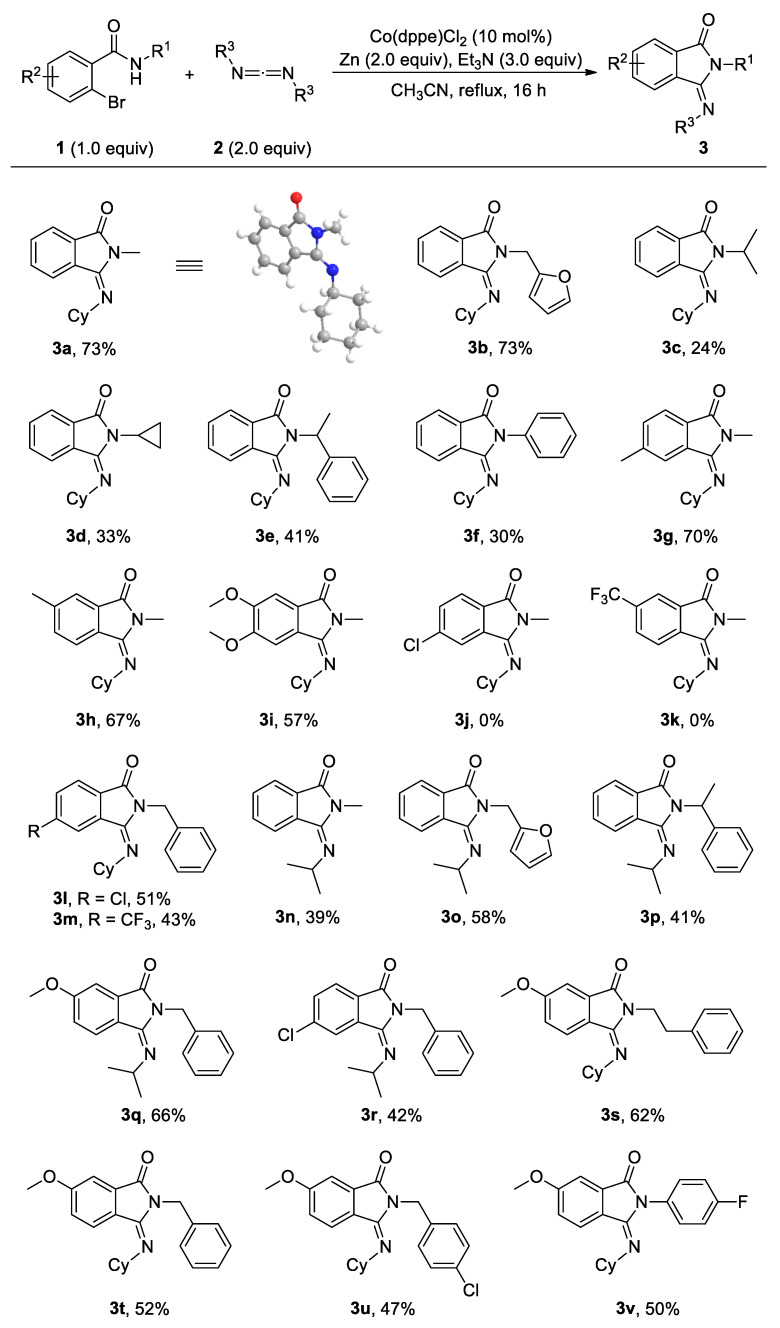
Reaction scope ^a,b^. ^a^ Condition: **1** (0.5 mmol, 1.0 equiv), **2** (1.0 mmol, 2.0 equiv), Co(dppe)Cl_2_ (10 mol%), Zn (2.0 equiv), Et_3_N (3.0 equiv), CH_3_CN (1.5 mL) reflux under N_2_ for 16 h. ^b^ Isolated yield.

We carried out the cobalt-catalyzed cyclization reaction of 2-bromobenzamides with alkynes [35], then attempted to replace alkynes with carbodiimides to investigate the reactivity of this replacement. Our study started from the optimization of the reaction conditions by using the model substrates 2-bromo-*N*-methylbenzamide (**1a**) and *N*,*N*’-dicyclohexylcarbodiimide (**2a**) with various cobalt catalysts and bases. The results are summarized in Table 1. We first surveyed the optimized condition of our previous reaction [35]; however, the desired product **3a** was obtained in only 47% yield (Entry 1). Although the reaction proceeded smoothly, this result was still unsatisfying and thus made us further investigate various factors of the conditions for this cobalt catalytic cyclization reaction. We then examined other cobalt catalysts (Entries 2–4) and found that Co(dppe)Cl_2_ had the best performance and could provide the desired product **3a** in 73% yield (Entry 3). Product **3a** was verified by ^1^H, ^13^C NMR, and high-resolution mass spectrometry, which was further determined as *E*-form by single-crystal X-ray analysis (see Supplementary Material for the spectral data and Scheme 3 for the X-ray structure). Additionally, the effect of the base was investigated. When we introduced other bases such as pyrolidine, pyridine, and K_2_CO_3_ to replace triethylamine, the yields of the desired product decreased significantly with organic bases (Entries 5 and 6), and the reaction did not proceed with K_2_CO_3_ (Entry 7). Next, we tested various solvents to check the reactivity of reactions in different solvents (Entries 8–13). It was found that the yields of product **3a** in other solvents were much worse than that in acetonitrile. We often observed the protonation of substrate **1a** as the major side product, except the reactions in DMSO. The crude ^1^H NMR spectra of the reactions in DMSO were wholly different from those in other solvents. We observed many unidentified side reactions in DMSO, and product **3a** could not be clearly isolated. An increase in reaction time was not helpful. When we increased the reaction time from 16 to 24 h, we obtained product **3a** in almost the same yield (Entry 14). The blank reactions were investigated as well, and the results indicate that the cobalt catalyst, the ligand, and the reducing agent zinc are key factors and are all necessary (Entries 15–17). The reaction could not proceed without any of them.

After obtaining the optimized reaction conditions, we then investigated the reaction scope by testing various substituents to understand the capacity of this cobalt-catalyzed cyclization reaction, as illustrated in Scheme 3. We first investigated the effect of substituents that attached on the amide group (**3a**–**3f**). As indicated, primary alkyl groups can provide much higher yields than secondary alkyl and phenyl groups. Thus, the products with *N*-methyl (**3a**) and *N*-methylenefuryl (**3b**) groups were obtained in 73% yields, but the products with *N*-isopropyl (**3c**), *N*-cyclopropyl (**3d**), *N*-1-phenylethyl (**3e**), and *N*-phenyl (**3f**) groups could be provided only in 24, 33, 41, and 30% yields, respectively. We next screened the substituents on the benzamide moiety (**3g**–**3m**) and found an interesting trend of the reaction. The reaction proceeded well for the electron-donating groups on the backbone phenyl moiety and obtained the corresponding products **3g**–**3i** in moderate yields. However, when an electron-withdrawing group was introduced to the *N*-methyl substrate, the reactions could not proceed well to form the desired products **3j** and **3k**. We further modified the *N*-protected groups from methyl to benzyl to increase the electron density of the amide nitrogen, and the corresponding products **3l** and **3m** could be obtained in 51 and 43% yields, respectively. These results clearly indicate that the lower electron density on the amide nitrogen led to the lower yields of the desired products.

We further changed the substituent on carbodiimide from the cyclohexyl group to the isopropyl group (**3n**–**3r**) to study the performance of this substrate in this cobalt catalytic cyclization reaction. It was found that *N*,*N*’-diisopropylcarbodiimide could also proceed with the reaction smoothly when the proper substituents were constructed on substrate **1**, and we could observe the same reaction trend through the yields of desired products. Products with the electron-donating groups on the nitrogen or the phenyl moiety (**3o** and **3q**) could provide higher yields than other structures (**3n**, **3p**, and **3r**). Moreover, we combined the methoxy group on the phenyl moiety with other *N*-attached substituents to establish various structures (**3s**–**3v**), since an electron-donating group is a guaranteed group to provide the products with comparably higher yields. The products were indeed obtained in moderate yields either with the *N*-alkyl or the *N*-aryl groups. Notably, comparing the spectral data of previous reports [5,6,7,8,9,10,11,12,13,14] with ours, as well as the single-crystal structure of **3a**, all products were determined as the *E*-form single isomer as their exact structures.

**Scheme 4:**
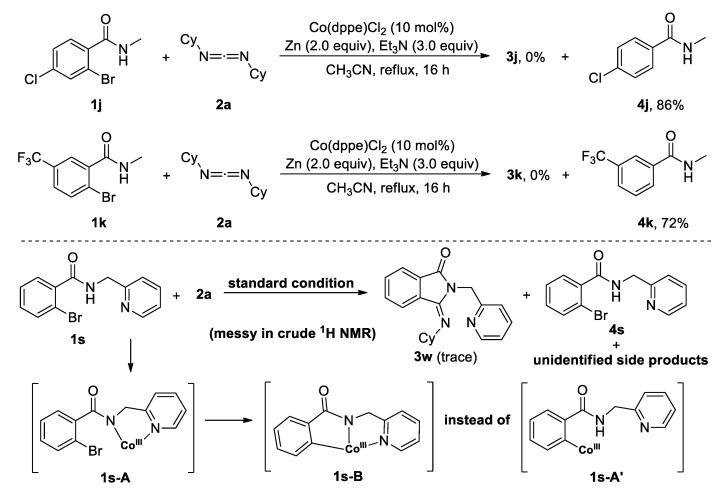
Control experiments.

From the results of the investigation of the substrate scope, we noticed that the reactions strongly rely on the electron density of benzamides. The reactions generally could proceed well only for the electron-rich substrates. For the substrates with an electron-withdrawing group on the backbone phenyl moiety, reactions did not provide satisfying yields or even be inhibited, especially for those with an *N*-methyl group. This aroused our curiosity and prompted us to study the reaction in detail. We then conducted several control experiments and tried to verify the reaction pathway from the results (Scheme 4). First, we carefully checked the reactions of the electron-deficient substrates **1j** and **1k** and found that the protonation of substrate **1** dominated the reactions. Compounds **4j** and **4k** were identified as major products in the reactions, and the recovered yields were very high. No other side reactions were found in these reactions. This implies that the activation of the C-Br bond in these two substrates is fast and clear, and no other competitive reaction can occur in the presence of **1j** and **1k**. However, when the substrate with an *N*-methylene-2-pyridinyl group (**1s**) was utilized in the reaction, the performance of the reaction was totally different. We obtained messy crude NMR spectra. The reaction was complicated, and no compound could be identified as the major product. Several compounds, including the desired product **3w**, the corresponding protonation product **4s**, and the unidentified side products, could be detected by GC-MS in poor yields. This result was probably caused by the formation of intermediate **1s-A**, which was supposed to be formed by the chelation of two nitrogen atoms on substrate **1s**. According to the reports, the formation of **1s-A** is favorable than that of **1s-A′** [11,12,13,19], and complex **1s-A** will further convert to **1s-B** via activation of a C-Br bond. Therefore, the different intermediates resulted in different reaction behavior.

**Scheme 5:**
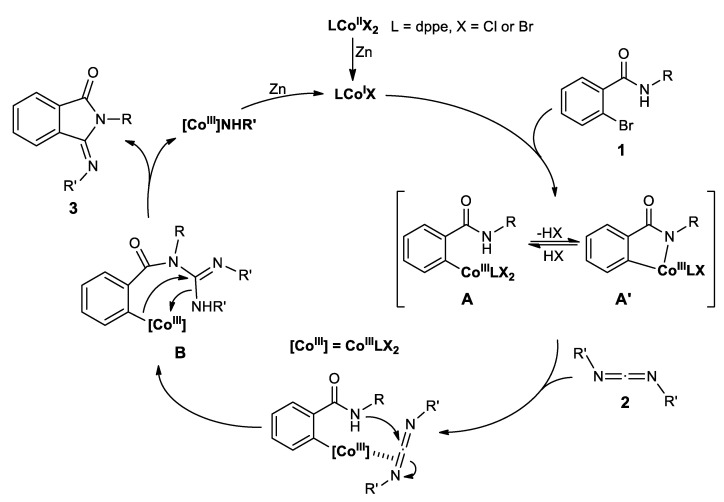
Proposed reaction mechanism.

Based on the above results and a previous report [35], we propose the reaction mechanism of this cobalt-catalyzed cyclization reaction as below (Scheme 5). The reaction is likely to be initiated by reduction of the Co(II) catalyst to form an active Co(I) species, which activates the C-Br bond of substrate **1** via oxidative addition or a two-times single-electron-transfer process to form the resulting Co(III) complex **A** [20]. An equilibrium between complexes **A** and **A′** will occur by the participation and departure of an HX molecule. Formation of the five-membered aza-cobalacycle complex **A′** can provide extra stability for this Co(III) species [11,12,13,14,15], and protonation of complex **A** will lead to the side product **4**. Substrate **2** then participates in the reaction via coordination with complex **A**, which causes the following nucleophilic addition of the amide group to the central carbon of carbodiimide to obtain another Co(III) complex **B**. The aryl moiety attached to the Co(III) species is able to perform as a nucleophile to attack the same carbon and generate product **3** with the Co(III) species [15]. Reduction of the resulting Co(III) species leads to the regeneration of the active Co(I) catalyst.

**Scheme 6:**
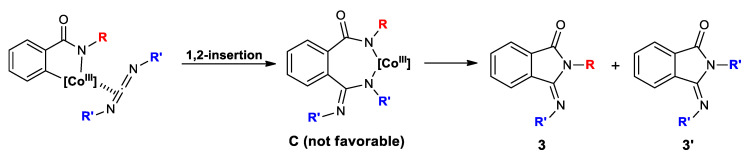
Alternative reaction pathway.

An alternative pathway should be described as proceeding the cyclization by the formation of complex **C** through the 1,2-insertion of carbodiimide (Scheme 6). However, this reaction pathway will provide product **3** and the undetected compounds **3′** via the nucleophilic substitution of nitrogen to the carbonyl group or the iminyl group, which does not match our results. We did not observe compounds **3′** in the present cobalt catalytic cyclization reaction. Therefore, the formation of complex **C** is not favorable under the current reaction protocol.

## 3. Materials and Methods

All reagents were purchased from Sigma-Aldrich (St. Louis, MO, USA), Alfa-Aesar (Haverhill, MA, USA), TCI (Tokyo, Japan), and Fisher-Acros (Loughborough, UK), which were used without further purification unless otherwise noted. All manipulations of oxygen- and moisture-sensitive materials were conducted with a standard Schlenk technique or in a glove box. Flash column chromatography was performed using silica gel (230–400 mesh). Analytical thin-layer chromatography (TLC) was performed on 60 F_254_ (0.25 mm) plates, and visualization was accomplished with UV light (254 and 354 nm) and/or an aqueous alkaline KMnO_4_ solution followed by heating. Proton and carbon nuclear magnetic resonance spectra (^1^H NMR and ^13^C NMR) were recorded on a Bruker 300 or Bruker 600 spectrometer with Me_4_Si or solvent resonance as the internal standard (^1^H NMR, Me_4_Si at 0 ppm, CDCl_3_ at 7.26 ppm, *d_6_*-DMSO at 2.49 ppm; ^13^C NMR, Me_4_Si at 0 ppm, CDCl_3_ at 77.0 ppm, *d_6_*-DMSO at 39.7 ppm). ^1^H NMR data are reported as follows: chemical shift, multiplicity (s = singlet, d = doublet, t = triplet, q = quartet, quint = quintet, sext = sextet, sept = septet, br = broad, m = multiplet), coupling constants (Hz), and integration. IR spectral data were recorded on a Bruker TENSOR 37 spectrometer (Bruker, Billerica, MA, USA). Melting points (mp) were determined using an SRS OptiMelt MPA100 (Stanford Research Systems, Sunnyvale, CA, USA). GC-MS data were obtained from the HP 5890 Series II GC/HP 5972 GC MASS Spectrometer System. High-resolution mass spectral data were obtained from MAT-95XL HRMS by using the ESI method.

## 4. Conclusions

In conclusion, we developed a cobalt catalysis method to approach 3-(imino)isoindolin-1-one derivatives via the cyclization reactions of 2-bromobenzamides with carbodiimides. This catalytic reaction demonstrated tolerance to diverse substituents and could provide the desired products in moderate yields for most cases. The reaction mechanism is proposed through the formation of a five-membered aza-cobalacycle, which proceeds the nucleophilic addition of amide to carbodiimide with subsequent C-C bond formation to generate the desired products. Further studies of other relative cobalt catalytic reactions, as well as their applications, are currently underway.

## Figures and Tables

**Table 1:**
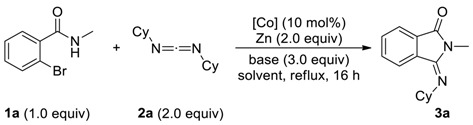
Optimization of reaction conditions ^a,b^.

Entry	[Co]	Base	Solvent	Yield (%) ^b^
1	Co(dppe)Br_2_	Et_3_N	CH_3_CN	47
2	Co(dppf)Br_2_	Et_3_N	CH_3_CN	26
3	Co(dppe)Cl_2_	Et_3_N	CH_3_CN	73
4	Co(dppf)Cl_2_	Et_3_N	CH_3_CN	17
5	Co(dppe)Cl_2_	pyrolidine	CH_3_CN	16
6	Co(dppe)Cl_2_	pyridine	CH_3_CN	21
7	Co(dppe)Cl_2_	K_2_CO_3_	CH_3_CN	0
8	Co(dppe)Cl_2_	Et_3_N	CH_2_Cl_2_	trace
9 ^c^	Co(dppe)Cl_2_	Et_3_N	DMF	13
10	Co(dppe)Cl_2_	Et_3_N	THF	23
11	Co(dppe)Cl_2_	Et_3_N	Ethanol	29
12	Co(dppe)Cl_2_	Et_3_N	DMSO	messy
13	Co(dppe)Cl_2_	Et_3_N	Toluene	39
14 ^c^	Co(dppe)Cl_2_	Et_3_N	CH_3_CN	72
15	CoCl_2_	Et_3_N	CH_3_CN	0
16 ^d^	Co(dppe)Cl_2_	Et_3_N	CH_3_CN	0
17	-	Et_3_N	CH_3_CN	0

^a^ Reaction conditions: **1a** (0.5 mmol, 1.0 equiv), **2a** (1.0 mmol, 2.0 equiv), [Co] (10 mol%), Zn (2.0 equiv), base (3.0 equiv), solvent (1.5 mL) reflux under N_2_ for 16 h. ^b^ Isolated yield. ^c^ 24 h reaction time. ^d^ Reaction without zinc.

## Data Availability

Not applicable.

## Supplementary Materials

The following are available online, experimental procedures, spectral data, X-ray Diffraction Analysis of Compound **3a**, ^1^H and ^13^C NMR spectra for products.

## Sample Availability

Samples of the compounds are not available from the authors.
