# Supplementary material for: Cobalt-Catalyzed Cyclization of 2-Bromobenzamides with Carbodiimides: A New Route for the Synthesis of 3-(Imino)isoindolin-1-ones
Source: Molecules. 2021 Nov 28;26(23):7212. doi: 10.3390/molecules26237212 (PMC8658922; doi:10.3390/molecules26237212)

Supporting Information

Cobalt-Catalyzed Cyclization of 2-Bromobenzamides with Carbodiimides: A New Route for the Synthesis of 3-(Imino)isoindolin-1-ones

Hasil Aman [1], Yu-Chiao Huang [1], Yu-Hao Liu [1], Yu-Lin Tsai [2], Min Kim [3], Jen-Chieh Hsieh [2,*] and Gary Jing Chuang [1,*]

[1] Department of Chemistry, Chung Yuan Christian University, Taoyuan, 320314, Taiwan (R.O.C.); hasilaman786@gmail.com (H. A.); jojohuang1234567890@gmail.com (Y.-C. H.); ytfdfvhhbbn@gmail.com (Y.-H. L.)
[2] Department of Chemistry, Tamkang University, New Taipei City, 251301, Taiwan (R.O.C.); zzz759153@gmail.com
[3] Department of Chemistry, Chungbuk National University, 28644, Korea; minkim@chungbuk.ac.kr
* Correspondence: jchsieh@mail.tku.edu.tw (J.C. H.); gjchuang@cycu.edu.tw (G. J. C.); Tel.: +886-2-2621-5656 ext 2545 (J.C. H.); Tel.: +886-3-2653312 (G. J. C.).

Table of Contents

Table of Contents	Page No
**General Information and procedure**	**S2**
**Spectral data for products**	**S3–S9**
**References**	**S9**
**X-ray data of 3a**	**S10**
**^{1}H and ^{13}C NMR spectra**	**S11–S31**

General information:

All reagents were purchased from Sigma-Aldrich (St. Louis, MO, USA), Alfa-Aesar (Haverhill, MA, USA), TCI (Tokyo, Japan) and Fisher-Acros (Loughborough, UK), which were used without further purification unless otherwise noted. All manipulations of oxygen- and moisture-sensitive materials were conducted with a standard Schlenk technique or in the glove box. Flash column chromatography was performed using silica gel (230-400 mesh). Analytical thin layer chromatography (TLC) was performed on 60 F_{254} (0.25 mm) plates and visualization was accomplished with UV light (254 and 354 nm) and/or an aqueous alkaline $KMnO_4$ solution followed by heating. Proton and carbon nuclear magnetic resonance spectra (^{1}H NMR and ^{13}C NMR) were recorded on Bruker 300 or Bruker 600 spectrometer with Me_4Si or solvent resonance as the internal standard (^{1}H NMR, Me_4Si at 0 ppm, $CDCl_3$ at 7.26 ppm, d_6-DMSO at 2.49 ppm; ^{13}C NMR, Me_4Si at 0 ppm, $CDCl_3$ at 77.0 ppm, d_6-DMSO at 39.7 ppm). ^{1}H NMR data are reported as follows: chemical shift, multiplicity (s = singlet, d = doublet, t = triplet, q = quartet, quint = quintet, sext = sextet, sept = septet, br = broad, m = multiplet), coupling constants (Hz), and integration. IR spectral data were recorded on a Bruker TENSOR 37 spectrometer (Bruker, Billerica, MA, USA). Melting points (mp) were determined using a SRS OptiMelt MPA100 (Stanford Research Systems, Sunnyvale, CA, USA). GC-MS data were obtained from the HP 5890 Series II GC/HP 5972 GC MASS Spectrometer System. High Resolution Mass spectral data were obtained from MAT-95XL HRMS by using ESI method. X-ray data was obtained from Bruker APEX DUO. Starting materials of various *N*-substituted 2-halobenzamides **1a-x** were synthesized according to the reported procedure.[1]

General procedure for the synthesis of 3-(imino)isoindolin-1-ones 3:

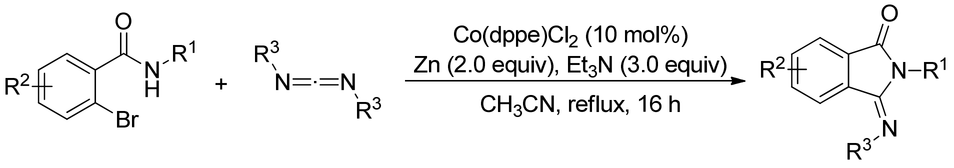

1 (1.0 equiv) **2** (2.0 equiv) **3**

A screw-capped vial (10-mL) was added substituted 2-bromobenzamide **1** (0.5 mmol, 1.0 equiv), carbodiimide **2** (1.0 mmol, 2.0 equiv), $Co(dppe)Cl_2$ (0.05 mmol, 10 mol%), Zn (1.0 mmol, 2.0 equiv) and NEt_3 (1.0 mmol, 2.0 equiv) in dry CH_3CN (1.5 mL). The septum fitted screw caped vial was then evacuated and purged with nitrogen for around 10 minutes and quickly replaced the septum by a Teflon screw cap. The mixture was then allowed to stir at 90 °C for 16 h. After the reaction complete, the reaction mixture was filtered through a short celite pad, which was then washed with dichloromethane for several times. The filtrate was concentrated and the residue was purified on a silica gel column using hexanes and ethyl acetate as eluent to give the desired 3-iminoisoindoli-1-ones **3**.

All structures were characterized by the HRMS, ^{1}H NMR and ^{13}C NMR spectra. Products **3a** was verified by single-crystal X-ray diffraction. Spectral data, melting point, IR data, HRMS data as well as the copies of ^{1}H NMR and ^{13}C NMR spectra for all compounds are listed below.

Spectral data for all products:

(*E*)-3-(Cyclohexylimino)-2-methylisoindolin-1-one (3a)

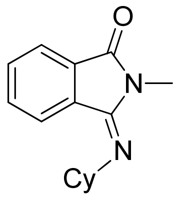

White solid; mp: 142–145 °C; ^{1}H NMR (400 MHz, $CDCl_3$): δ 7.92 (d, *J* = 6.8 Hz, 1H), 7.86 (d, *J* = 7.2 Hz, 1H), 7.63 (quint, *J* = 7.2 Hz, 2H), 4.27–4.20 (m, 1H), 3.25 (s, 3H), 1.91 (d, *J* = 9.3 Hz, 2H), 1.74 (d, *J* = 12.7 Hz, 3H), 1.52–1.34 (m, 5H); ^{13}C NMR (101 MHz, $CDCl_3$): δ 167.6, 149.6, 133.4, 132.7, 131.2, 129.8, 125.3, 123.3, 57.6, 34.6, 29.7, 25.7, 24.8; IR (KBr): 2926, 2367, 1733, 1662, 1655, 1494, 1378, 1090, 697 cm^{-1}; HRMS [(ESI), $(M+Na)^+$]: 265.1318 (cal. for $C_{15}H_{18}N_2NaO$ 265.1317).

(*E*)-3-(Cyclohexylimino)-2-(furan-2-ylmethyl)isoindolin-1-one (3b)

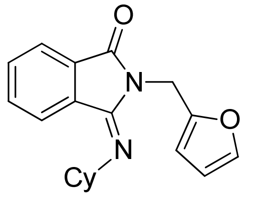

Colorless oil; ^{1}H NMR (400 MHz, $CDCl_3$): δ 7.89 (q, *J* = 12.2 Hz, 2H), 7.67–7.60 (m, 2H), 7.30 (d, *J* = 10.7 Hz, 1H), 4.99 (s, 1H), 4.25 (t, *J* = 9.5 Hz, 1H), 1.89 (d, *J* = 10.0 Hz, 4H), 1.67 (q, *J* = 13.4 Hz, 2H), 1.34–1.21 (m, 7H); ^{13}C NMR (101 MHz, $CDCl_3$): δ 167.7, 143.4, 132.0, 130.9, 130.3, 126.0, 123.2, 57.5, 34.5, 29.7, 25.7, 24.7, 22.3; IR (KBr): 3116, 2918, 2810, 2362, 2214, 1725, 1659, 1509, 1363, 1220, 1144, 701 cm^{-1}**;** HRMS [(ESI), $(M+Na)^+$]: 331.1426 (cal. for $C_{19}H_{20}N_2NaO_2$ 331.1422).

(*E*)-3-(Cyclohexylimino)-2-isopropylisoindolin-1-one (3c)

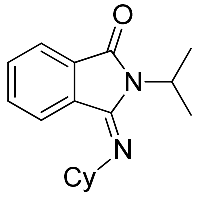

White solid; mp 129–131 °C**;** ^{1}H NMR (400 MHz, $CDCl_3$): δ 7.87 (q, *J* = 6.8 Hz, 2H), 7.63 (t, *J* = 6.4 Hz, 2H), 4.82 (br, 1H), 4.29–4.24 (m, 1H), 1.92 (d, *J* = 6.8 Hz, 4H), 1.70 (t, *J* = 8.0 Hz, 6H), 1.51 (d, *J* = 6.9 Hz, 3H), 1.51 (d, *J* = 7.2 Hz, 3H); ^{13}C NMR (101 MHz, $CDCl_3$): δ 167.4, 139.9, 138.2, 133.3, 131.0, 129.5, 127.5, 119.2, 55.7, 42.2, 34.9, 25.7, 24.5, 22.7; IR (KBr): 2923, 2362, 2219, 1722, 1647, 1512, 1460, 1426, 1362, 700 cm^{-1}; HRMS [(ESI), $(M+Na)^+$]: 293.1626 (cal. for $C_{17}H_{22}N_2NaO$ 293.1630).

(*E*)-3-(Cyclohexylimino)-2-cyclopropylisoindolin-1-one (3d)

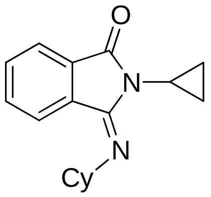

White solid; mp 129–131 °C**;** ^{1}H NMR (400 MHz, $CDCl_3$): δ 7.89–7.83 (m, 2H), 7.64–7.60 (m, 2H), 4.25 (t, *J* = 9.6 Hz, 1H), 2.80 (s, 1H), 1.91 (d, *J* = 10.8 Hz, 4H), 1.71–1.65 (m, 2H), 1.49–1.43 (m, 4H), 1.04–1.03 (m, 4H); ^{13}C NMR (101 MHz, $CDCl_3$): δ 167.5, 148.3, 133.5, 132.5, 131.0, 129.5, 125.2, 123.1, 57.2, 51.2, 43.1, 34.5, 29.4, 26.3, 25.8, 25.5, 24.5 19.9; IR (KBr): 2920, 2822, 2363, 2219, 1723, 1642, 1510, 1461, 1429, 1362, 1103, 697 cm^{-1}; HRMS [(ESI), $(M+Na)^+$]: 291.1473 (cal. for $C_{17}H_{20}N_2NaO$ 291.1473).

(*E*)-3-(Cyclohexylimino)-2-(1-phenylethyl)isoindolin-1-one (3e)

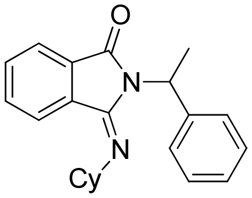

Colorless oil**;** ^{1}H NMR (400 MHz, $CDCl_3$): δ 7.88 (d, *J* = 6.4 Hz, 1H), 7.84 (d, *J* = 7.2 Hz, 1H), 7.60 (quint t, *J* = 7.6, 1.2 Hz, 2H), 7.54 (d, *J* = 7.6 Hz, 2H), 7.31 (t, *J* = 7.2 Hz, 2H), 7.23 (t, *J* = 7.2 Hz, 1H), 5.89 (q, *J* = 7.6 Hz, 1H), 4.29–4.23 (m, 1H), 1.97 (d, *J* = 7.2 Hz, 3H), 1.92–1.82 (m, 6H), 1.49–1.38 (m, 4H); ^{13}C NMR (101 MHz, $CDCl_3$): δ 167.4, 147.9, 141.7, 133.3, 132.7, 131.1, 129.4, 127.9, 127.7, 126.9, 125.3, 123.3, 57.1, 49.2, 34.5, 34.3, 29.4, 25.8, 24.3, 17.2; IR (KBr): 3001, 2971, 2806, 2362, 2220, 1739, 1648, 1492, 1381, 1222, 1109, 703 cm^{-1}; HRMS [(ESI), $(M+Na)^+$]: 355.1785 (cal. for $C_{22}H_{24}N_2NaO$ 355.1786).

(*E*)-3-(Cyclohexylimino)-2-phenylisoindolin-1-one (3f)

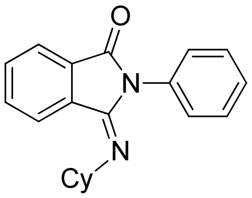

Light yellow solid; mp 122–125°C; ^{1}H NMR (400 MHz, $CDCl_3$): δ 8.03 (dd, *J* = 7.2, 1.2 Hz, 1H), 7.97 (d, *J* = 7.6 Hz, 1H), 7.74 (td, *J* = 7.6, 1.2 Hz, 1H), 7.69 (td, *J* = 7.6, 1.2 Hz, 1H), 7.50–7.44 (m, 4H), 7.38–7.34 (m, 1H), 4.33–4.28 (m, 1H), 1.91–1.82 (m, 4H), 1.71–1.68 (m, 2H), 1.49–1.33 (m, 4H); ^{13}C NMR (101 MHz, $CDCl_3$): δ 166.9, 148.8, 134.0, 133.3, 132.7, 131.6, 129.5, 128.6, 128.4, 128.2, 127.2, 125.6, 123.9, 57.7, 34.2, 29.7, 25.6, 24.6**;** IR (KBr): 2964, 2822, 2362, 2219**,** 1726, 1662, 1367, 1140, 697 cm^{-1}**;** HRMS [(ESI), $(M+Na)^+$]: 327.1473 (cal. for $C_{20}H_{20}N_2NaO$ 327.1473).

(*E*)-3-(Cyclohexylimino)-2,5-dimethylisoindolin-1-one (3g)

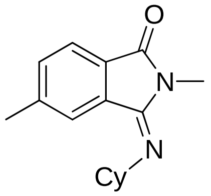

White solid; mp 102–105 °C; ^{1}H NMR (400 MHz, $CDCl_3$): δ 7.76 (d, *J* = 8.0 Hz, 1H), 7.61 (s, 1H), 7.39 (dd, *J* = 2.7, 0.4 Hz, 1H), 4.22–4.17 (m, 1H), 3.20 (s, 3H), 2.50 (s, 3H), 1.90–1.87 (m, 4H), 1.61–1.58 (m, 2H), 1.47–1.36 (m, 4H); ^{13}C NMR (100 MHz, $CDCl_3$): δ 167.7, 143.4, 131.9, 130.9, 130.2, 125.9, 123.1, 57.5, 34.5, 29.7, 25.7, 25.2, 24.7, 22.3; IR (KBr): 2967, 2354, 1721, 1653, 1518, 1396, 703 cm^{-1}; HRMS [(ESI), $(M+Na)^+$]: 279.1476 (cal. for $C_{16}H_{20}N_2NaO$ 279.1473).

(*E*)-3-(Cyclohexylimino)-2,6-dimethylisoindolin-1-one (3h)

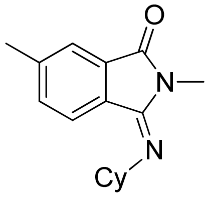

White solid; mp 105–107 °C; ^{1}H NMR (400 MHz, $CDCl_3$): δ 7.73 (d, *J* = 8.2 Hz, 2H), 7.44 (s, *J* = 7.8 Hz, 1H), 4.19 (m, 1H), 3.23 (s, 3H), 2.50 (s, 3H), 1.90 (t, *J* = 5.1 Hz, 4H), 1.73 (t, *J* = 6.1 Hz, 2H), 1.46 (q, *J* = 4.2 Hz, 4H); ^{13}C NMR (101 MHz, $CDCl_3$): δ 167.8, 149.6, 141.9, 133.7, 133.2, 127.3, 125.1, 123.7, 57.6, 34.6, 25.7, 25.1, 24.8, 21.6; IR (KBr): 2962, 2344, 2212, 1724, 1613, 1521, 1398, 700 cm^{-1}; HRMS [(ESI), $(M+Na)^+$]: 279.1475 (cal. for $C_{16}H_{20}N_2NaO$ 279.1473).

(*E*)-3-(Cyclohexylimino)-5,6-dimethoxy-2-methylisoindolin-1-one (3i)

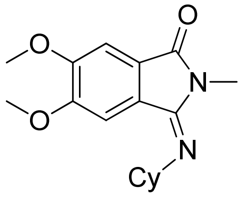

White solid; mp 65–68 °C; ^{1}H NMR (400 MHz, $CDCl_3$): δ 7.38 (s, 1H), 7.33 (s, 1H), 4.17–4.10 (m, 1H), 4.00 (d, *J* = 5.0 Hz, 6H), 3.21 (s, 3H), 1.94 (d, *J* = 10.3 Hz, 4H), 1.74 (t, *J* = 4.9 Hz, 2H), 1.45–1.34 (m, 4H); ^{13}C NMR (101 MHz, $CDCl_3$): δ 167.9, 156.8, 152.4, 127.0, 123.0, 108.0, 105.2, 57.8, 56.3, 49.1, 34.6, 34.0, 25.6, 25.0; IR (KBr): 2927, 2880, 2361, 2202, 1726, 1648, 1498, 1382, 1236, 1116, 701 cm^{-1}; HRMS [(ESI), $(M+Na)^+$]: 325.1573 (cal. for $C_{17}H_{22}N_2NaO_3$ 325.1528).

(*E*)-2-benzyl-5-chloro-3-(cyclohexylimino)isoindolin-1-one (3l)

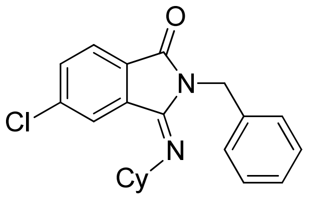

White solid; mp 143–146 °C ; ^{1}H NMR (400 MHz, $CDCl_3$): δ 7.83 (t, J = 9.5 Hz, 2H), 7.58 (d, J = 8.0 Hz, 1H), 7.48 (d, J = 7.4 Hz, 2H), 7.31–7.24 (m, 3H), 4.97 (s, 2H), 4.19 (q, J = 6.2 Hz, 1H), 1.88 (d, J = 8.9 Hz, 4H), 1.73–1.63 (m, 3H), 1.49–1.39 (m, 3H); ^{13}C NMR (101 MHz, $CDCl_3$): δ 166.3, 139.0, 137.6, 131.5, 131.1, 129.0, 128.3, 127.3, 125.7, 124.6, 57.3, 42.1, 34.4, 25.7, 24.2; IR (KBr): 2926, 2365, 2215, 1734, 1660, 1493, 1376, 1091, 702 cm^{-1}; HRMS [(ESI), (M+Na)$^+$]: 375.1236 (cal. for $C_{21}H_{21}ClN_2NaO$ 375.1240).

(*E*)-2-Benzyl-3-(cyclohexylimino)-5-(trifluoromethyl)isoindolin-1-one (3m)

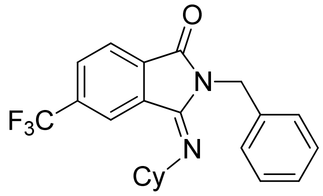

Colorless oil**;** ^{1}H NMR (400 MHz, $CDCl_3$): δ 8.07 (s, 1H), 8.05 (d, J = 8.0 Hz, 1H), 7.90 (d, J = 8.0 Hz, 1H), 7.49 (t, J = 7.2 Hz, 2H), 7.32–7.25 (m, 3H), 5.01 (s, 2H), 4.28–4.23 (m, 1H), 1.91–1.88 (m, 4H), 1.74–1.63 (m, J = 5.9 Hz, 4H), 1.49 (q, J = 9.0 Hz, 4H); ^{13}C NMR (101 MHz, $CDCl_3$): δ 165.8, 146.7, 137.4, 136.1, 134.7(q, $J_{C\text{-}F}$ = 32.5 Hz), 129.9, 129.0, 128.5(q, $J_{C\text{-}F}$ = 3.7 Hz), 128.3, 127.4, 124.0, 123.4 (q, $J_{C\text{-}F}$ = 271.5 Hz), 122.3 (q, $J_{C\text{-}F}$ = 4.3 Hz), 57.6, 42.3, 34.4, 29.7, 25.6, 24.2; IR (KBr): 3068, 2916, 1750, 1674, 1521, 1410, 1384, 1009, 810 cm^{-1}; HRMS [(ESI), (M+Na)$^+$]: 409.1529 (cal. for $C_{22}H_{21}F_3N_2NaO$ 409.1504).

(*E*)-3-(Isopropylimino)-2-methylisoindolin-1-one (3n)

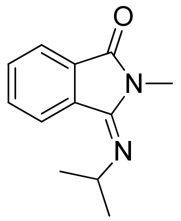

White solid; mp 130–132°C; ^{1}H NMR (400 MHz, $CDCl_3$): δ 7.92 (t, J = 8.2 Hz, 2H), 7.66–7.59 (m, 2H), 4.67–4.61 (m, 1H), 3.24 (s, 3H), 1.37 (d, J = 6.2 Hz, 6H); ^{13}C NMR (101 MHz, $CDCl_3$): δ 167.6, 149.4, 133.9, 133.4, 132.6, 131.2, 129.9, 125.4, 123.3, 49.5, 25.1, 24.6; IR (KBr): 2926, 2366, 1721, 1648, 1515, 1462, 1426, 1363, 698 cm^{-1}; HRMS [(ESI), (M+Na)$^+$]: 225.1007 (cal. for $C_{12}H_{14}N_2NaO$ 225.1004).

(*E*)-2-(Furan-2-ylmethyl)-3-(isopropylimino)isoindolin-1-one (3o)

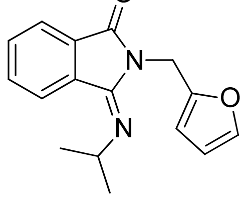

White solid; mp 76–78 °C; ^{1}H NMR (400 MHz, $CDCl_3$): δ 7.94 (q, J = 2.9 Hz, 2H), 7.67–7.60 (m, 2H), 7.32 (s, 1H), 6.30 (q, J = 3.3 Hz, 2H), 5.01 (s, 2H), 4.67–4.60 (m, 1H), 1.37 (d, J = 6.2 Hz, 6H); ^{13}C NMR (101 MHz, $CDCl_3$): δ 166.9, 151.0, 141.7, 133.1, 132.9, 131.4, 129.8, 125.5, 123.7, 110.2, 108.2, 49.6, 34.9, 29.7, 24.5; IR (KBr): 3110, 2922, 2823, 2362, 2219, 1723, 1655, 1503, 1366, 1222, 1138, 700 cm^{-1}; HRMS [(ESI), (M+Na)$^+$]: 291.1106 (cal. for $C_{16}H_{16}N_2NaO_2$

291.1109).

(*E*)-3-(Isopropylimino)-2-(1-phenylethyl)isoindolin-1-one (3p)

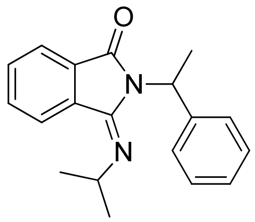

Colorless oil; ^{1}H NMR (400 MHz, $CDCl_3$): δ 7.93–7.86 (m, 2H), 7.64–7.53 (m, 4H), 7.29 (d, *J* = 8.3 Hz, 2H), 7.22 (t, *J* = 7.3 Hz, 1H), 5.88 (q, *J* = 7.3 Hz, 1H), 4.66–4.59 (m, 1H), 1.95 (d, *J* = 7.3 Hz, 3H), 1.28 (s, 6H); ^{13}C NMR (101 MHz, $CDCl_3$): δ 167.9, 156.8, 152.4, 127.0, 123.0, 108.0, 105.2, 57.8, 56.3, 49.1, 34.6, 34.0, 25.6, 25.0; IR (KBr): 2971, 2934, 2806, 2352, 2218, 1739, 1703, 1673, 1489, 1440, 1377, 1202, 1109 cm^{-1}; HRMS [(ESI), (M+Na)$^{+}$]: 315.1473 (cal. for $C_{19}H_{20}N_2NaO$ 315.1473).

(*E*)-2-Benzyl-3-(isopropylimino)-6-methoxyisoindolin-1-one (3q)

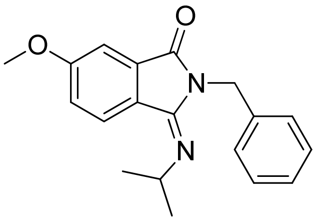

Colorless oil; ^{1}H NMR (400 MHz, $CDCl_3$): δ 7.82 (d, *J* = 8.6 Hz, 1H), 7.49 (d, *J* = 7.2 Hz, 2H), 7.39 (d, *J* = 2.5 Hz, 1H), 7.31–7.23 (m, 3H), 7.11 (dd, *J* = 8.4, 2.4 Hz, 1H), 4.98 (s, 2H), 4.58–4.52 (m, 1H), 3.91 (s, 3H), 1.34 (d, *J* = 6.2 Hz, 6H); ^{13}C NMR (101 MHz, $CDCl_3$): δ 167.1, 162.1, 138.0, 135.6, 129.0, 128.2, 119.5, 107.2, 55.9, 49.5, 41.9, 29.7, 24.5; IR (KBr): 2925, 2844, 2367, 2220, 1745, 1653, 1491, 1380, 1233, 1110, 914, 697 cm^{-1}; HRMS [(ESI), (M+Na)$^{+}$]: 331.1422 (cal. for $C_{19}H_{20}N_2NaO_2$ 331.1422).

(*E*)-2-Benzyl-5-chloro-3-(isopropylimino)isoindolin-1-one (3r)

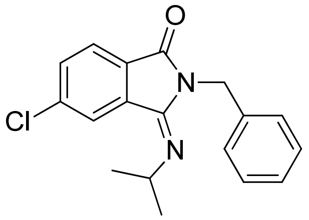

White solid; mp 47–49 °C ; ^{1}H NMR (400 MHz, $CDCl_3$): δ 7.94–7.91 (m, 2H), 7.66–7.59 (m, 2H), 7.51 (d, *J* = 7.1 Hz, 2H), 7.32–7.22 (m, 3H), 5.00 (s, 1H), 4.67–4.61 (m, 1H), 1.37 (d, *J* = 6.2 Hz, 6H); ^{13}C NMR (101 MHz, $CDCl_3$): δ 167.2, 137.9, 133.2, 132.8, 131.4, 129.8, 129.0, 128.2, 127.2, 125.5, 123.5, 49.6, 41.9, 29.7, 24.5, 22.7; IR (KBr): 2930, 2926, 2866, 2360, 2223, 1751, 1658, 1495, 1388, 1236, 1107, 698 cm^{-1}; HRMS [(ESI), (M+Na)$^{+}$]: 335.0928 (cal. for $C_{18}H_{17}ClN_2NaO$ 335.0927).

(*E*)-3-(Cyclohexylimino)-6-methoxy-2-phenethylisoindolin-1-one (3s)

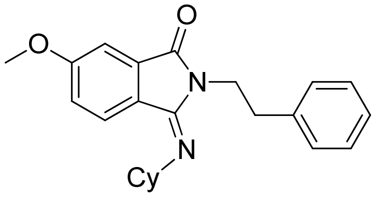

Colorless oil**;** ^{1}H NMR (400 MHz, $CDCl_3$): δ 7.75 (d, *J* = 8.6 Hz, 1H), 7.38 (d, *J* = 2.5 Hz, 1H), 7.29 (d, *J* = 4.4 Hz, 4H), 7.21 (q, *J* = 4.4 Hz, 1H), 7.13 (q, *J* = 3.7 Hz, 1H), 4.19–4.15 (m, 1H), 4.03 (t, *J* = 7.7 Hz, 2H), 3.92 (s, 3H), 3.03 (t, *J* = 7.7 Hz, 2H), 1.89 (t, *J* = 4.1 Hz, 4H), 1.73 (q, *J* = 5.2 Hz, 2H), 1.65–1.57 (m, 2H), 1.51–1.38 (m, 2H); ^{13}C NMR (101 MHz, $CDCl_3$): δ 167.1, 162.0, 148.0, 139.3, 135.6, 129.0, 128.3, 122.2, 119.4, 107.1, 57.2, 55.8, 39.9, 34.5, 25.8, 24.5; IR (KBr): 3070, 2949, 2860, 2362, 1736, 1658, 1470, 1382, 1239, 1115, 699 cm^{-1}; HRMS [(ESI), $(M+Na)^+$]: 385.1852 (cal. for $C_{23}H_{26}N_2NaO_2$ 385.1892).

(*E*)-2-Benzyl-3-(cyclohexylimino)-6-methoxyisoindolin-1-one (3t)

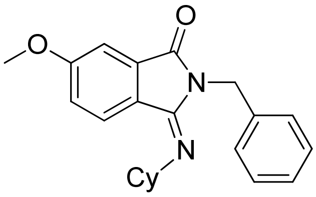

White solid; mp 110–112 °C ; ^{1}H NMR (400 MHz, $CDCl_3$): δ 7.74 (d, *J* = 8.5 Hz, 1H), 7.50 (t, *J* = 4.2 Hz, 2H), 7.39 (d, *J* = 2.5 Hz, 1H), 7.31–7.23 (m, 3H), 7.24 (d, *J* = 7.2 Hz, 1H), 7.12 (q, *J* = 3.7 Hz, 1H), 4.98 (s, 2H), 4.20–4.15 (m, 1H), 3.91 (s, 3H), 1.88 (q, *J* = 3.9 Hz, 4H), 1.71–1.58 (m, 2H), 1.49–1.29 (m, 3H); ^{13}C NMR (101 MHz, $CDCl_3$): δ 167.2, 162.1, 148.0, 138.0, 135.6, 129.0, 128.2, 127.1, 126.6, 122.3, 119.5, 107.2, 57.3, 55.8, 41.9, 34.5, 25.8, 24.5; IR (KBr): 3012, 2964, 2832, 2224, 1723, 1649, 1507, 1344, 1218, 1162, 1143, 702 cm^{-1}**;** HRMS [(ESI), $(M+Na)^+$]: 371.1732. (cal. for $C_{22}H_{24}N_2NaO_2$ 371.1735).

(*E*)-2-(4-Chlorobenzyl)-3-(cyclohexylimino)-6-methoxyisoindolin-1-one (3u)

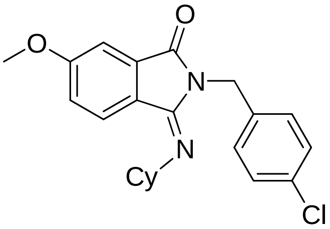

White solid; mp 66–68 °C; ^{1}H NMR (400 MHz, $CDCl_3$): δ 7.75 (d, *J* = 8.6 Hz, 1H), 7.38 (d, *J* = 2.5 Hz, 1H), 7.29 (d, *J* = 4.4 Hz, 3H), 7.21 (q, *J* = 4.4 Hz, 1H), 7.13 (q, *J* = 3.7 Hz, 1H), 4.17–4.10 (m, 1H), 4.03 (t, *J* = 7.7 Hz, 2H), 3.92 (s, 3H), 1.89 (t, *J* = 4.1 Hz, 4H), 1.73 (q, *J* = 5.2 Hz, 2H), 1.44–1.39 (m, 4H); ^{13}C NMR (101 MHz, $CDCl_3$): δ 167.1, 162.0, 148.0, 139.3, 135.6, 129.0, 128.3, 122.2, 119.4, 107.1, 57.2, 55.8, 39.9, 34.5, 25.8, 24.5; IR (KBr): 2927, 2866, 2360, 2221, 1746, 1658, 1498, 1382, 1236, 1117, 699 cm^{-1}; HRMS [(ESI), $(M+Na)^+$]: 405.1341 (cal. for $C_{22}H_{23}ClN_2NaO$ 405.1346).

(*E*)-3-(cyclohexylimino)-2-(4-fluorophenyl)-6-methoxyisoindolin-1-one (3v)

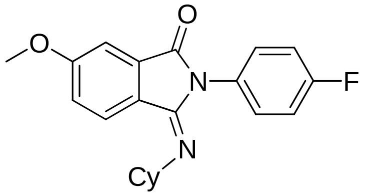

Light yellow solid; mp 134–136 °C; ^{1}H NMR (400 MHz, $CDCl_3$): δ 7.85 (d, *J* = 8.6 Hz, 1H), 7.48 (d, *J* = 2.5 Hz, 1H), 7.44–7.40 (m, 2H), 7.21 (d, *J* = 2.5 Hz, 1H), 7.16 (q, *J* = 5.8 Hz, 2H), 4.23 (t, *J* = 4.7 Hz, 1H), 3.97 (s, 3H), 1.89–1.81 (m, 4H), 1.69 (t, *J* = 6.0 Hz, 2H), 1.55–1.42 (m, 4H); ^{13}C NMR (101 MHz, $CDCl_3$): δ 166.8, 162.3, 161.4 (d, $J_{C\text{-}F}$ = 244.6 Hz), 148.7, 134.9, 129.8 (d, $J_{C\text{-}F}$ = 7.6 Hz), 127.0, 122.0, 120.4, 115.3 (d, $J_{C\text{-}F}$ = 22.5 Hz), 107.3, 57.6, 56.0, 34.2, 29.7, 25.6, 24.6; IR (KBr): 2966, 2830, 2362, 2214, 1725, 1659, 1509, 1363, 1220, 1140, 700 cm^{-1}**;** HRMS [(ESI), $(M+Na)^+$]: 375.1486 (cal. for $C_{21}H_{21}FN_2NaO_2$ 375.1485).

4-Chloro-*N*-methylbenzamide (4j)

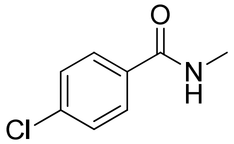

White solid; mp 166–168 °C; ^{1}H NMR (400 MHz, $CDCl_3$): δ 7.72 (d, *J* = 8.5 Hz, 2H), 7.41 (q, *J* = 3.6 Hz, 2H), 6.34 (s, 1H), 3.02 (q, *J* = 2.3 Hz, 3H); ^{13}C NMR (101 MHz, $CDCl_3$): δ 167.2, 137.6, 133.0, 128.8, 128.3, 26.9; IR (KBr): 3371, 3182, 2762, 1918, 1816, 1693, 1671, 1406, 1301, 1014 cm^{-1}**;** HRMS [(ESI), $(M+Na)^+$]: 192.0190 (cal. for $C_8H_8ClNNaO$ 192.0192).

N-Methyl-3-(trifluoromethyl)benzamide (4k)

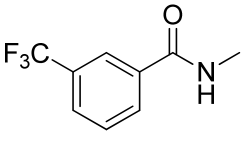

White solid; mp 174–176 °C; ^{1}H NMR (400 MHz, $CDCl_3$): δ 8.03 (s, 1H), 7.96 (d, *J* = 7.8 Hz, 1H), 7.73 (d, *J* = 7.8 Hz, 1H), 7.54 (t, *J* = 7.8 Hz, 1H), 6.66 (s, 1H), 3.01 (d, *J* = 4.8 Hz, 3H); ^{13}C NMR (101 MHz, $CDCl_3$): δ 166.9, 135.4, 131.2, 130.3, 129.2, 128.0, 123.9, 27.0; IR (KBr): 3388, 2376, 2920, 2851, 1672, 1622, 1422, 1321, 1141, 870 cm^{-1}**;** HRMS [(ESI), $(M+Na)^+$]: 226.0452 (cal. for $C_9H_8F_3NNaO$ 226.0456).

References

(1) Abe T.; Takahashi Y.; Matsubara Y.; Yamada K. *Org. Chem. Front.* **2017**, *4*, 2124.

Single-Crystal X-Ray Diffraction Analysis:

X-Ray Structure of Compound 3a:

(CCDC 2120041 (**3a**) contains the supplementary crystallographic data for this paper. These data can be obtained free of charge from The Cambridge Crystallographic Data Centre via www.ccdc.cam.ac.uk/data_request/cif.)

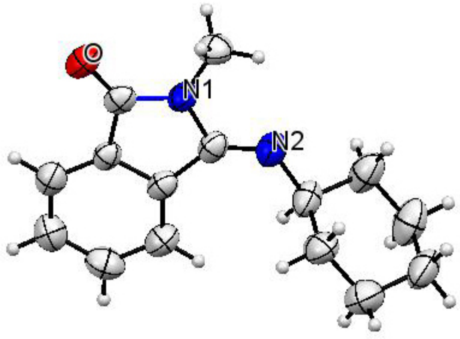

Figure S1. X-ray crystal structure of **3a**. Ellipsoids are drawn at the 50% probability level.

Table S1. Crystal data and structure refinement for **3a**.

Identification code	12311	
Empirical formula	C15H18N2O	
Formula weight	242.31	
Temperature	296(2) K	
Wavelength	0.71073 Å	
Crystal system	Monoclinic	
Space group	$P2_1/c$	
Unit cell dimensions	a = 13.006(8) Å	a= 90°.
	b = 14.967(6) Å	b= 102.59(4)°.
	c = 6.987(3) Å	g = 90°.
Volume	1327.3(11) Å^3	
Z	4	
Density (calculated)	1.213 Mg/m^3	
Absorption coefficient	0.077 mm^{-1}	
F(000)	520	
Crystal size	0.400 x 0.200 x 0.200 mm^3	
Theta range for data collection	1.604 to 28.365°.	
Index ranges	-17<=h<=14, -19<=k<=17, -9<=l<=9	
Reflections collected	9432	
Independent reflections	3255 [R(int) = 0.0865]	
Completeness to theta = 25.242°	99.7 %	
Absorption correction	None	
Refinement method	Full-matrix least-squares on F^2	
Data / restraints / parameters	3255 / 0 / 164	
Goodness-of-fit on F^2	1.014	
Final R indices [I>2sigma(I)]	R1 = 0.0627, wR2 = 0.1296	
R indices (all data)	R1 = 0.2041, wR2 = 0.1728	
Extinction coefficient	0.008(2)	
Largest diff. peak and hole	0.227 and -0.166 e.Å^{-3}	

^{1}H and ^{13}C NMR Spectra for Products (400 MHz, $CDCl_3$)

(*E*)-3-(Cyclohexylimino)-2-methylisoindolin-1-one (3a)

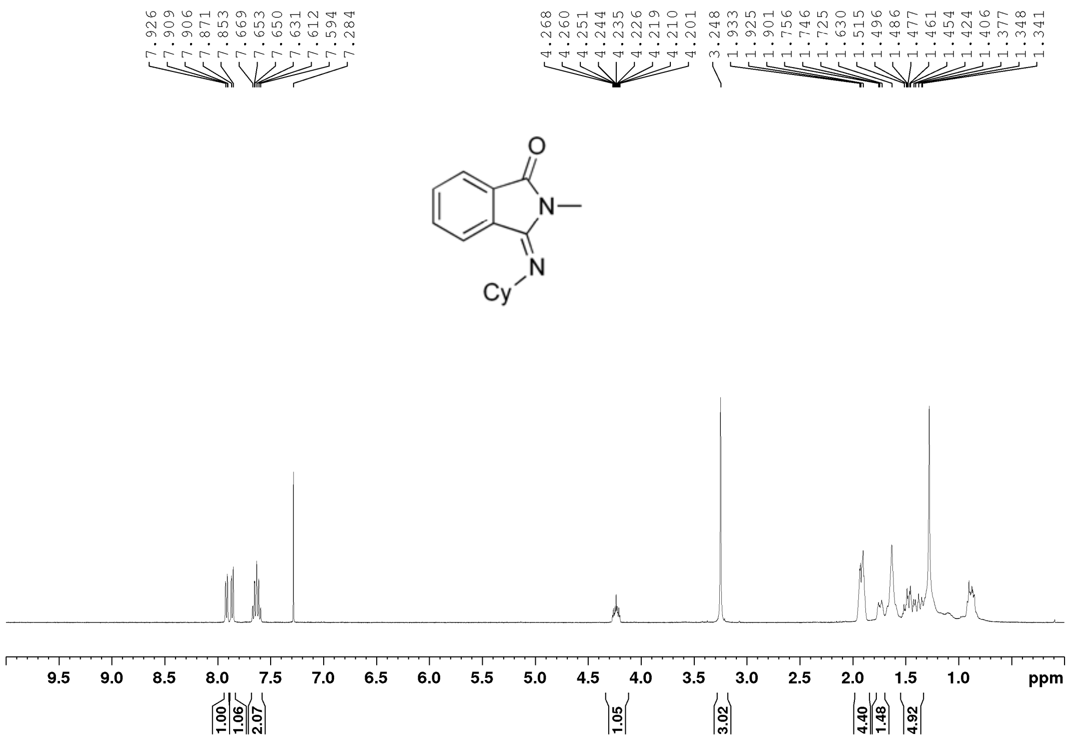

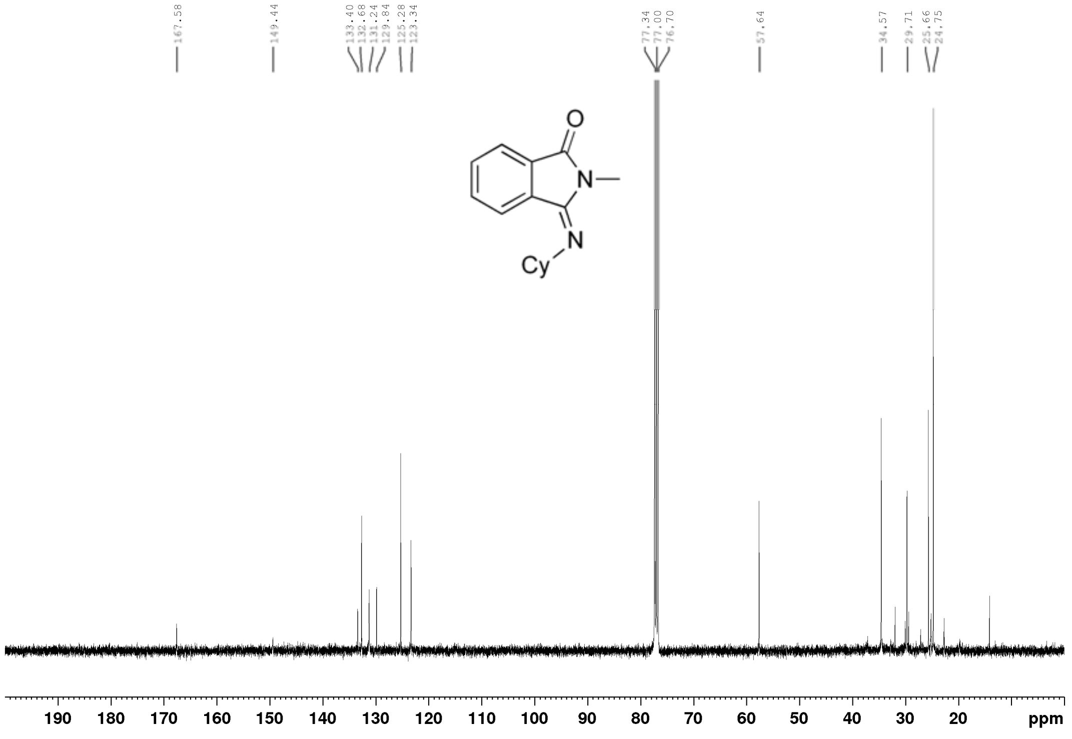

(*E*)-3-(Cyclohexylimino)-2-(furan-2-ylmethyl)isoindolin-1-one (3b)

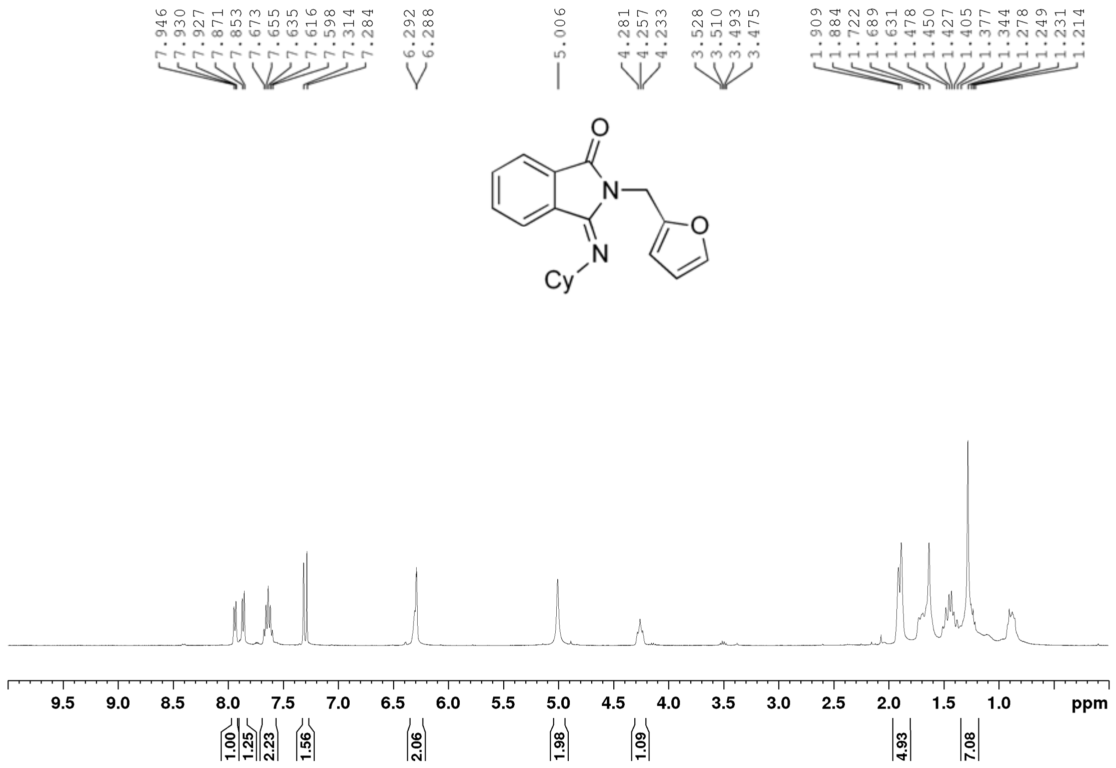

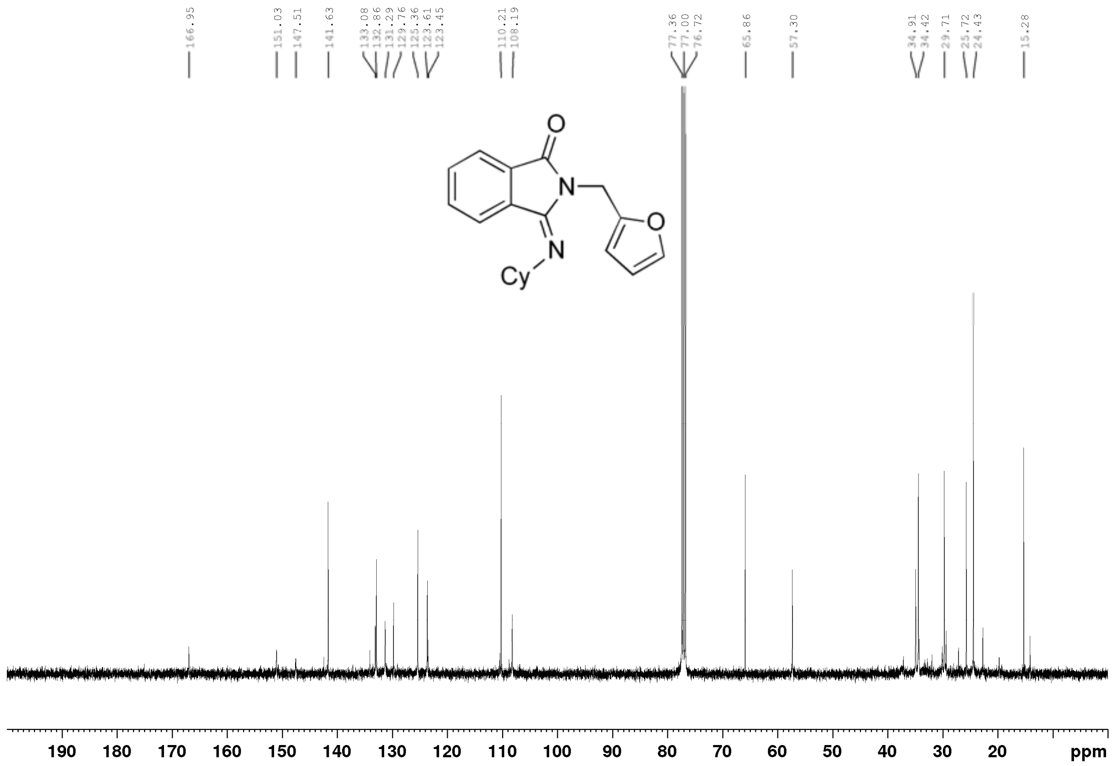

(*E*)-3-(Cyclohexylimino)-2-isopropylisoindolin-1-one (3c)

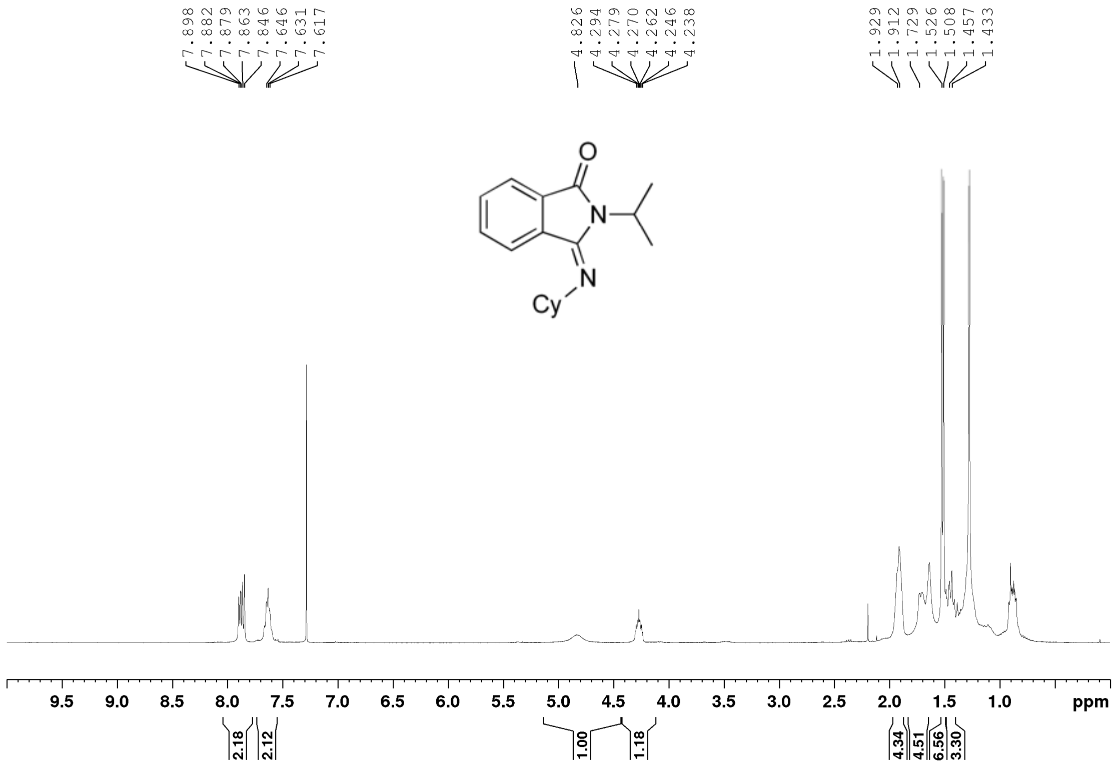

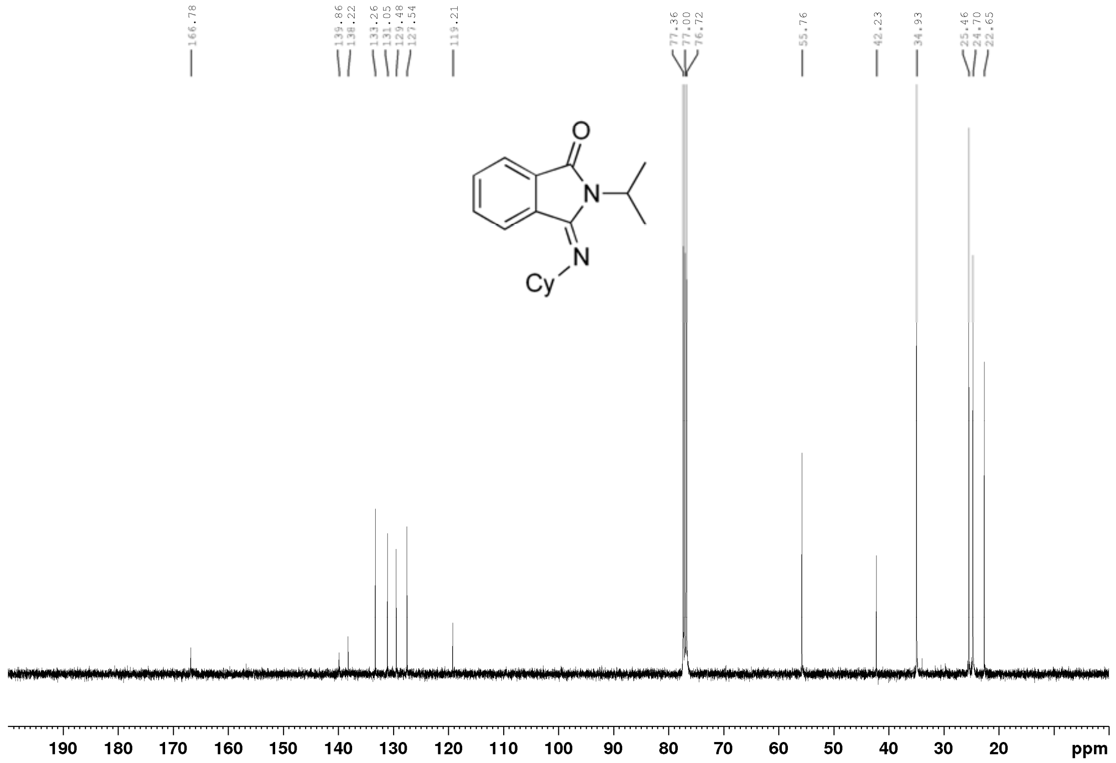

(*E*)-3-(Cyclohexylimino)-2-cyclopropylisoindolin-1-one (3d)

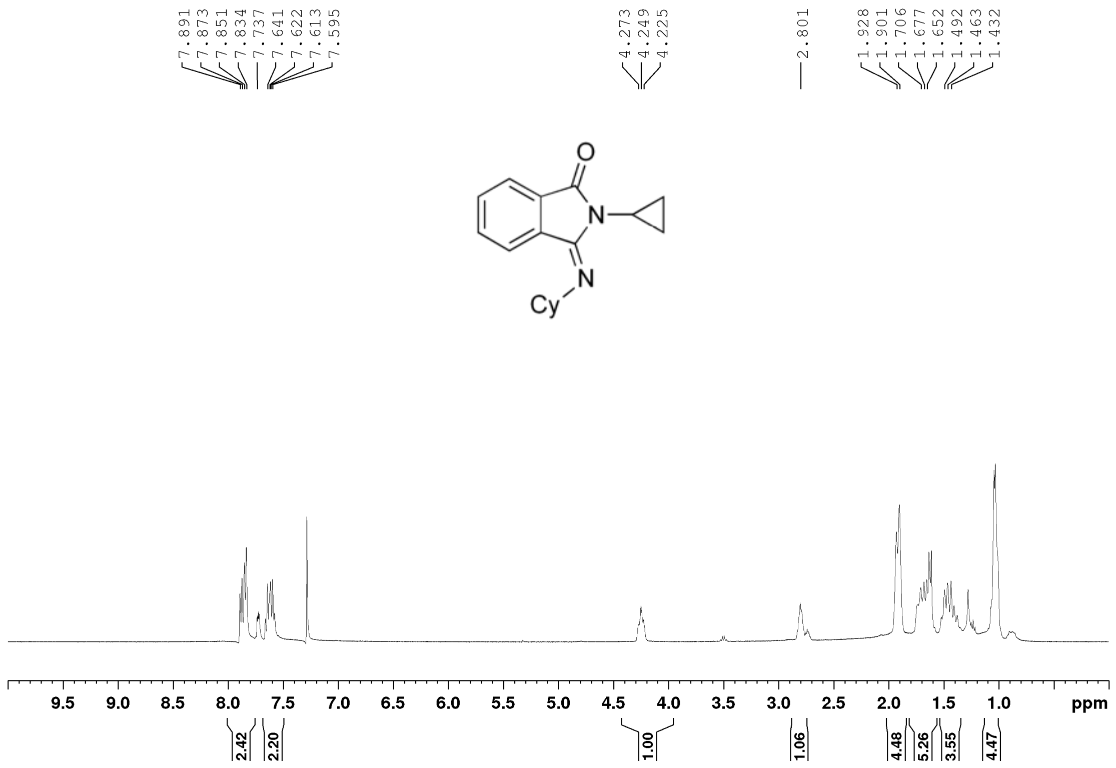

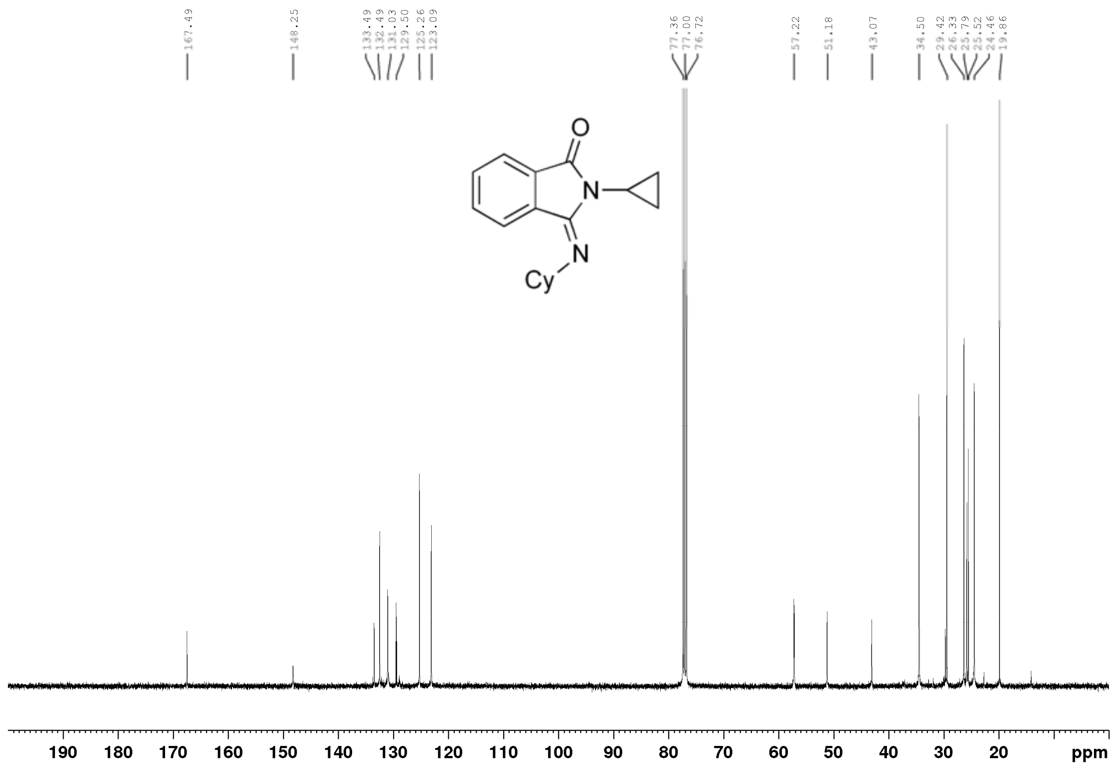

(*E*)-3-(Cyclohexylimino)-2-(1-phenylethyl)isoindolin-1-one (3e)

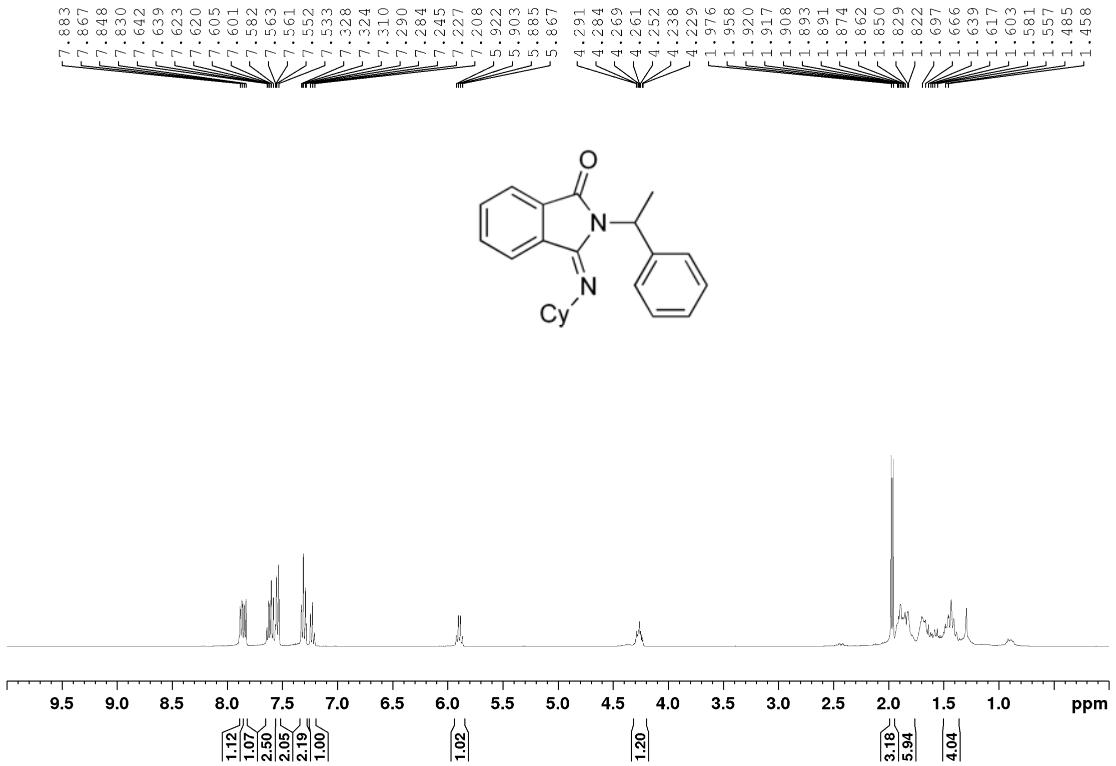

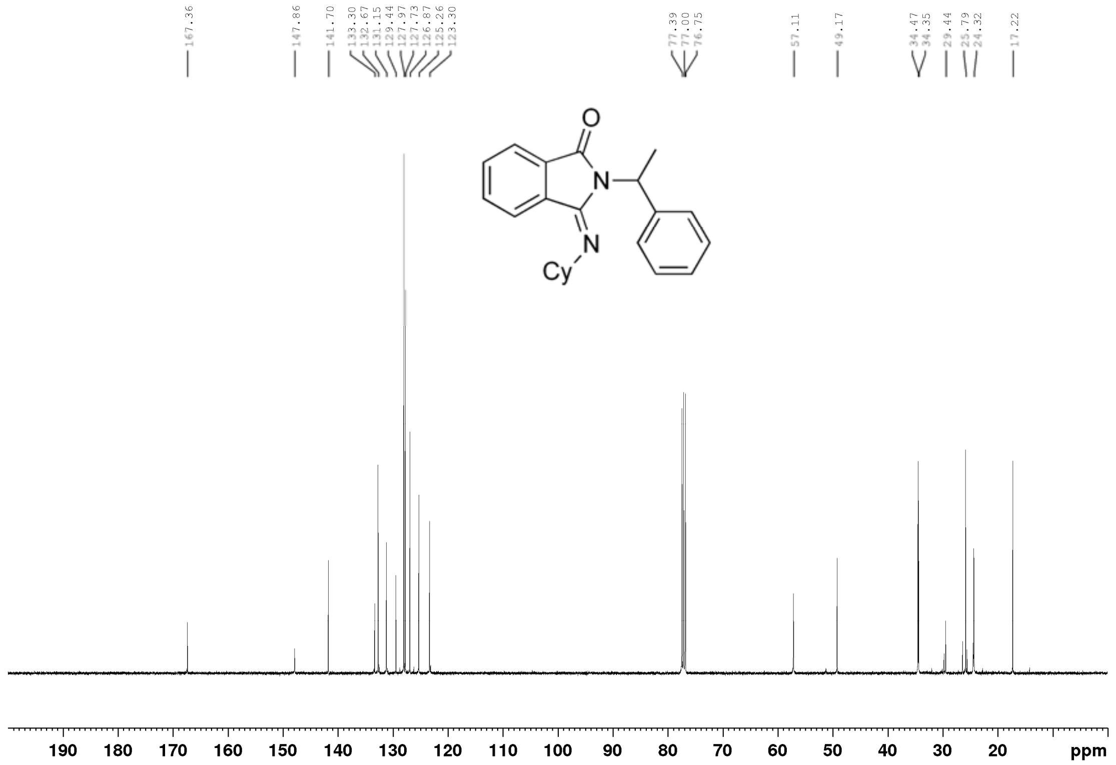

(*E*)-3-(Cyclohexylimino)-2-phenylisoindolin-1-one (3f)

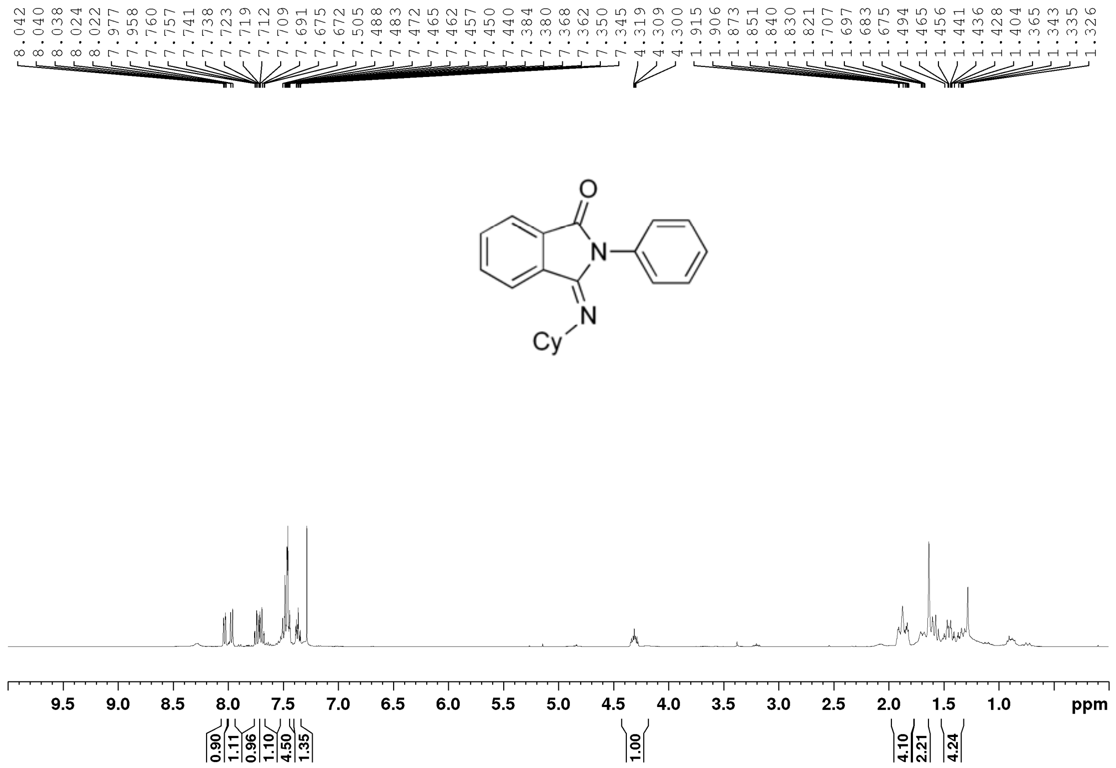

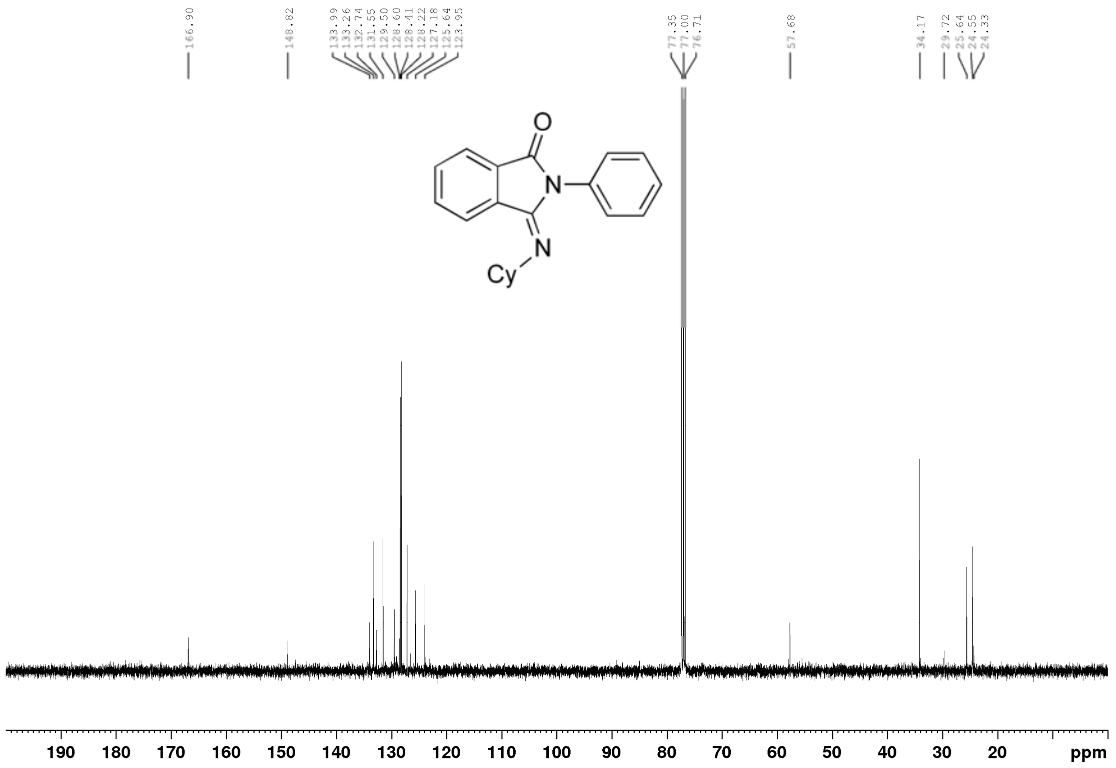

(*E*)-3-(Cyclohexylimino)-2,5-dimethylisoindolin-1-one (3g)

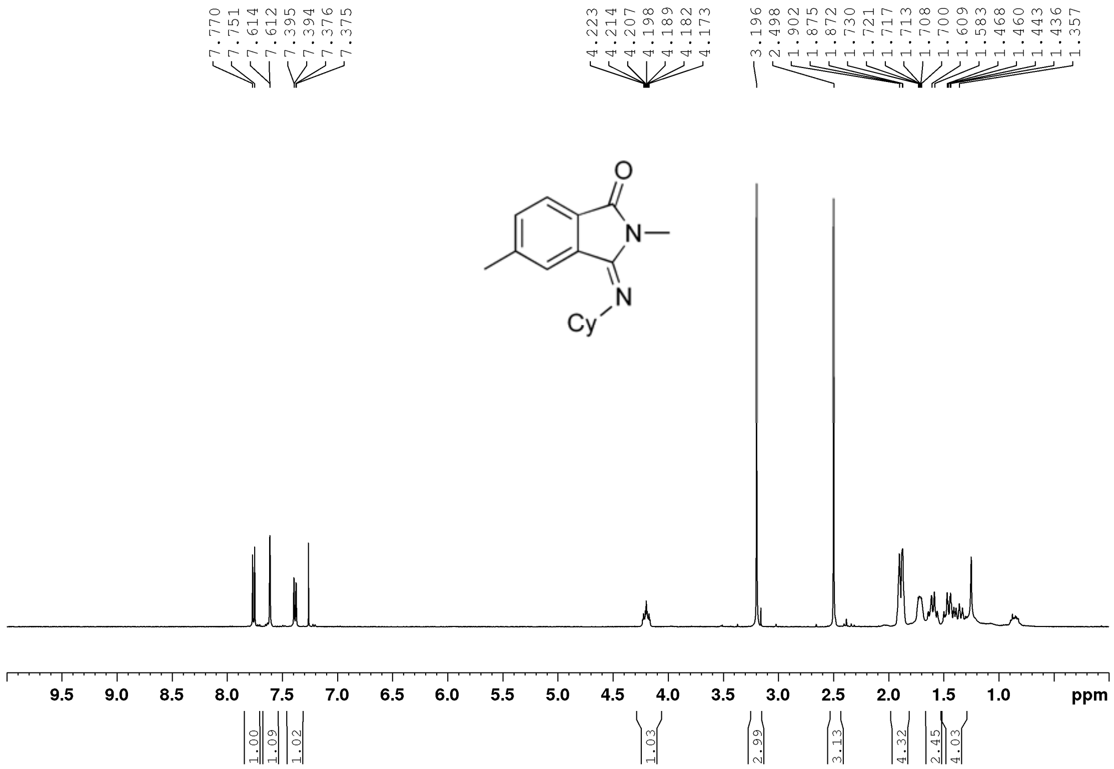

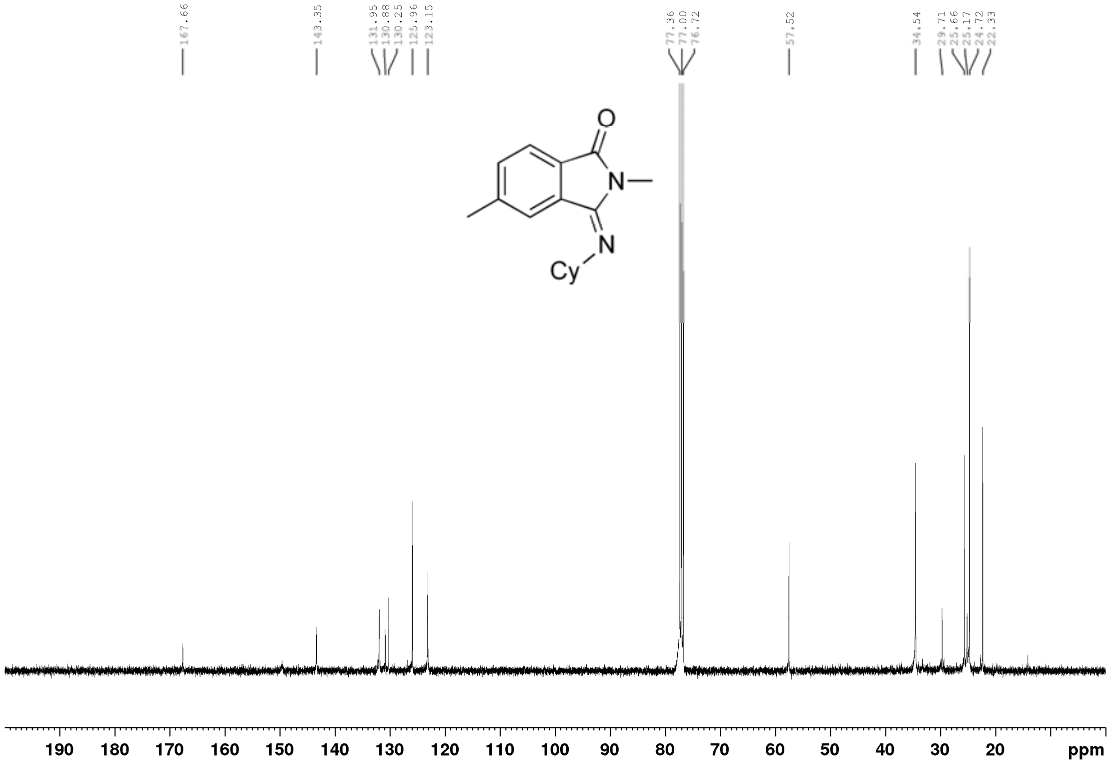

(*E*)-3-(Cyclohexylimino)-2,6-dimethylisoindolin-1-one (3h)

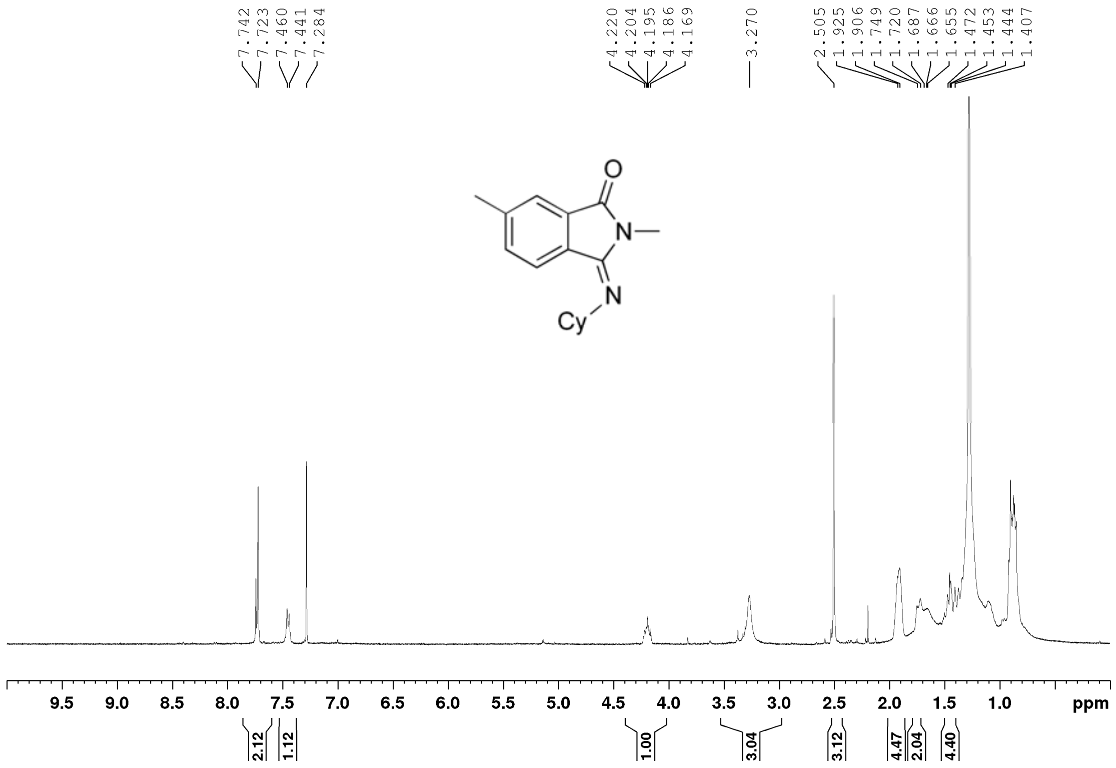

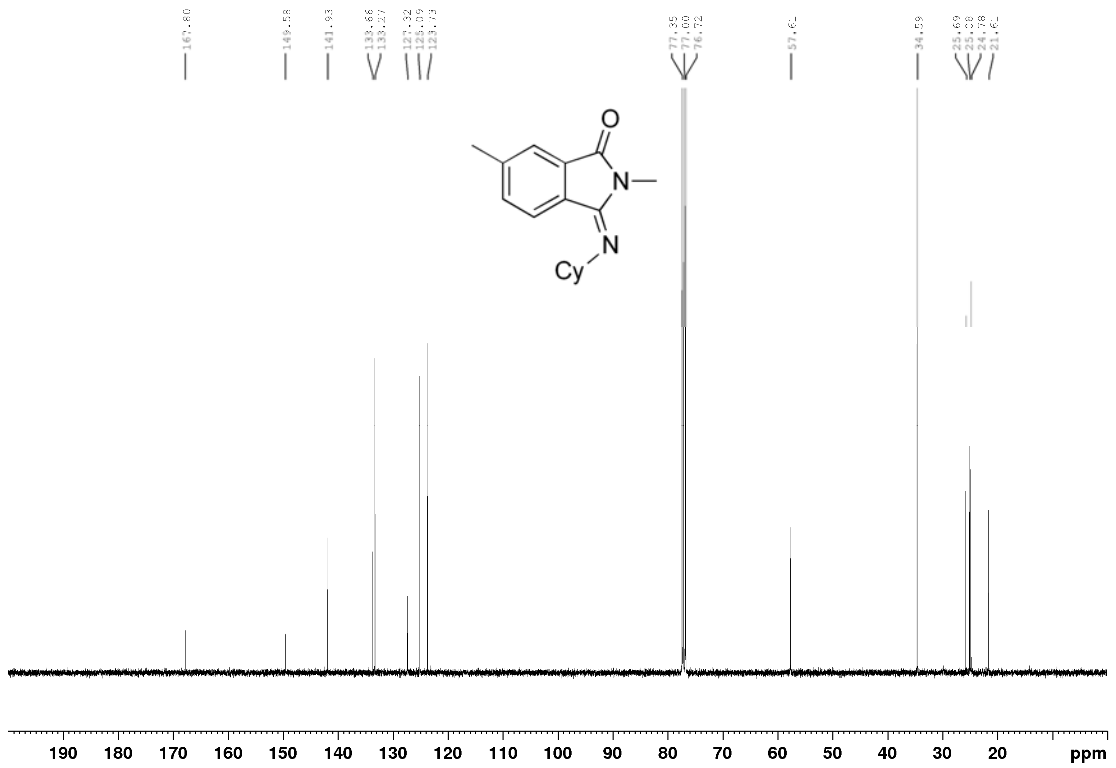

(*E*)-3-(Cyclohexylimino)-5,6-dimethoxy-2-methylisoindolin-1-one (3i)

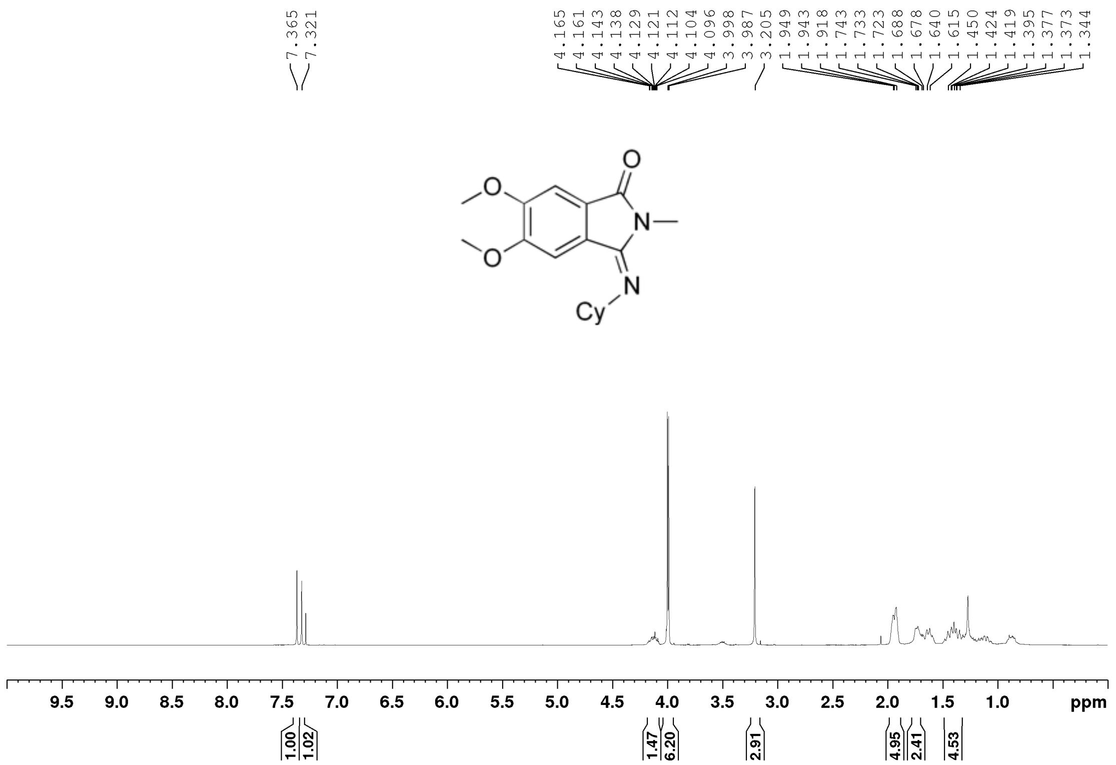

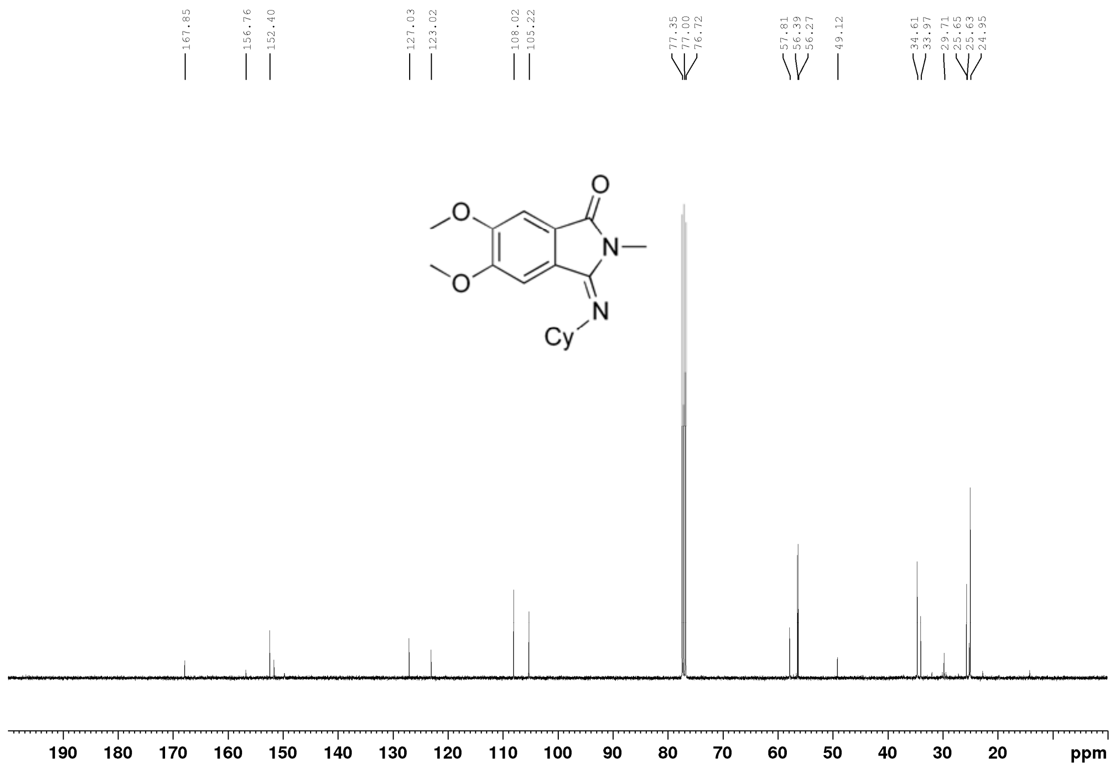

(*E*)-2-benzyl-5-chloro-3-(cyclohexylimino)isoindolin-1-one (3l)

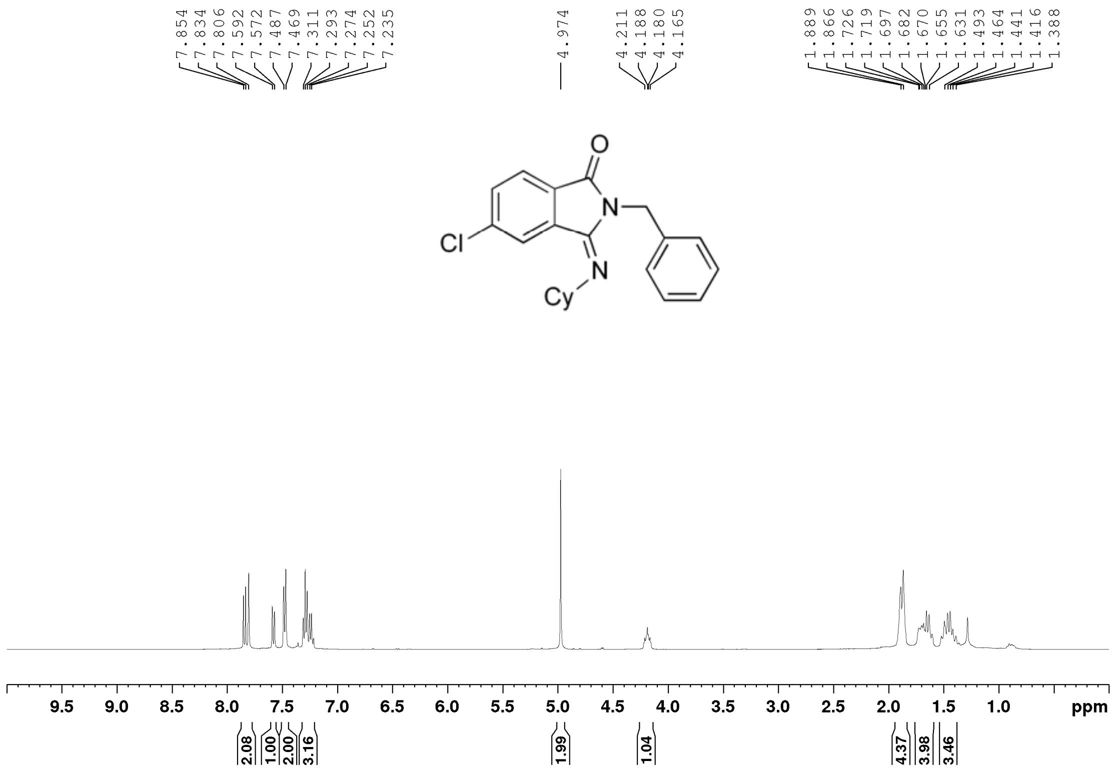

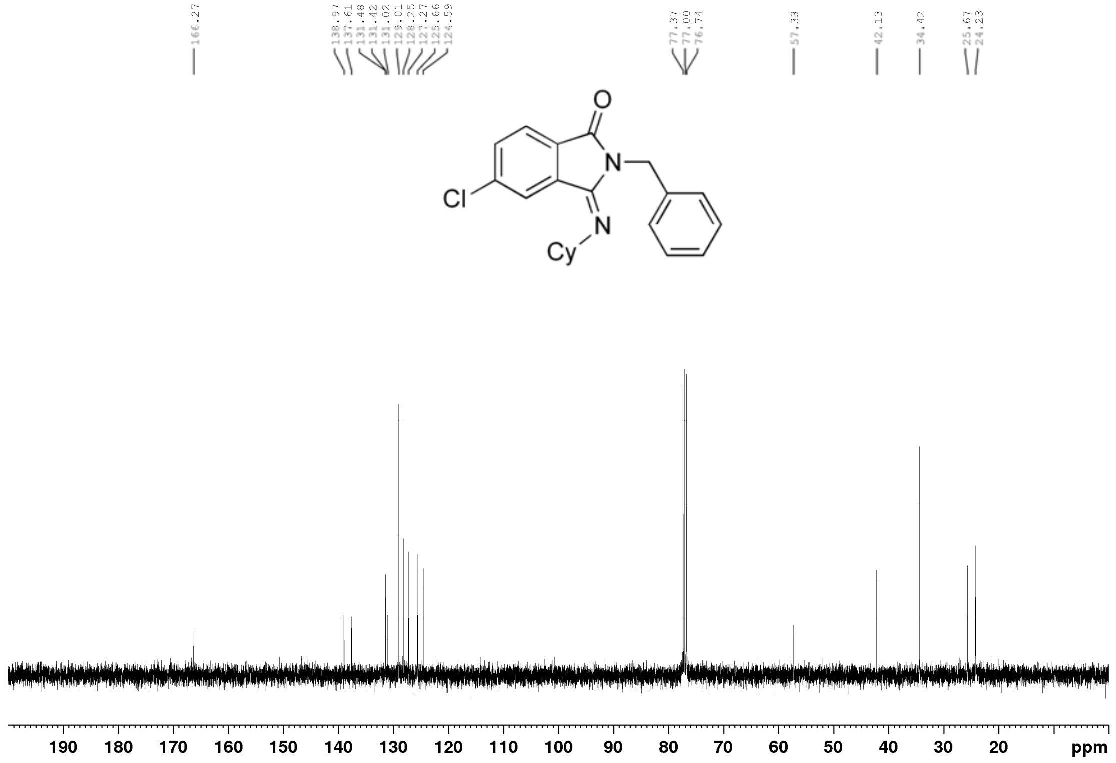

(*E*)-2-Benzyl-3-(cyclohexylimino)-5-(trifluoromethyl)isoindolin-1-one (3m)

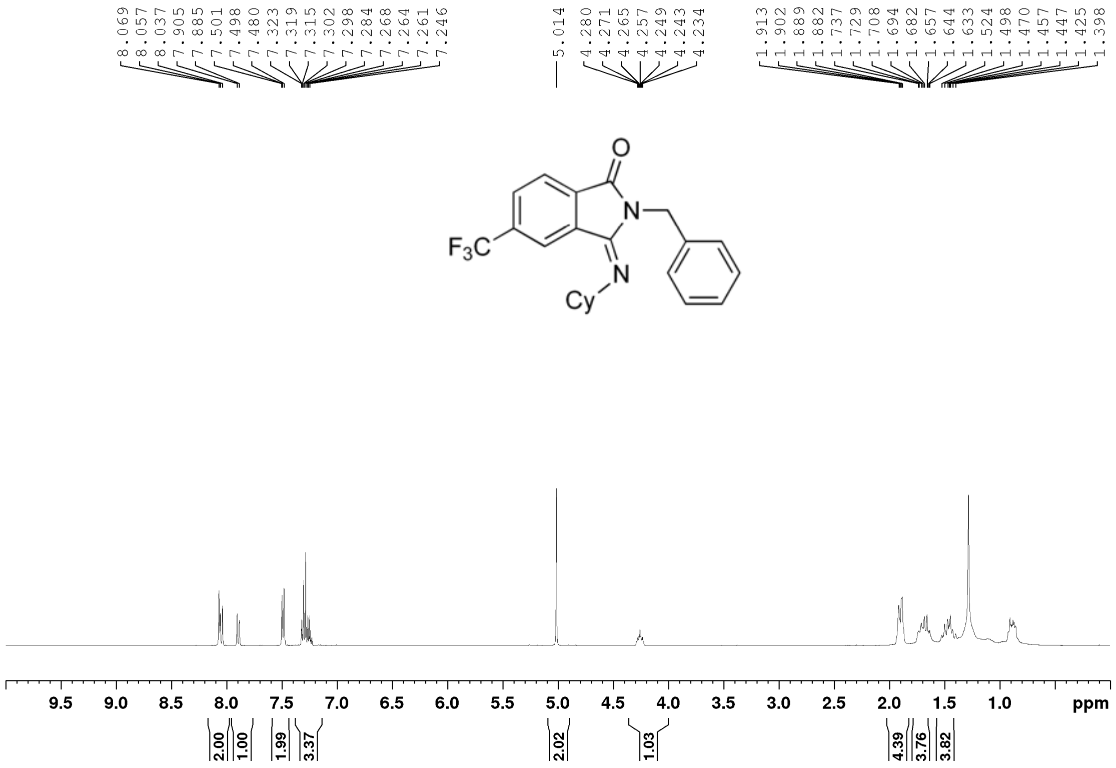

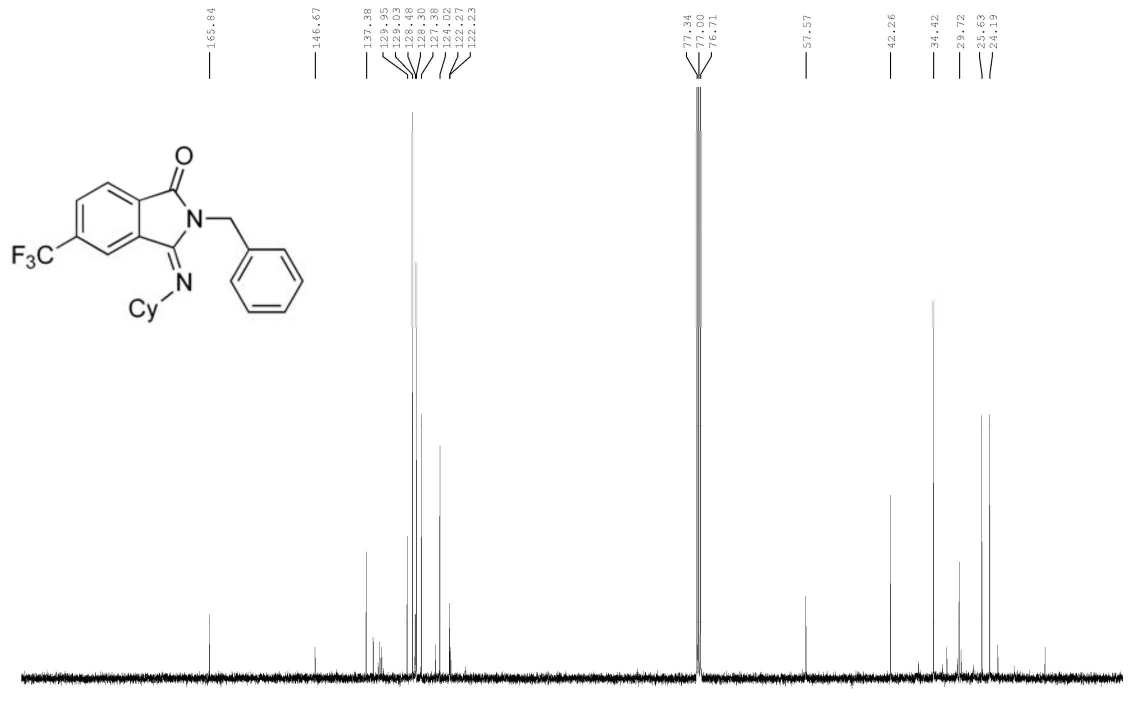

(*E*)-3-(Isopropylimino)-2-methylisoindolin-1-one (3n)

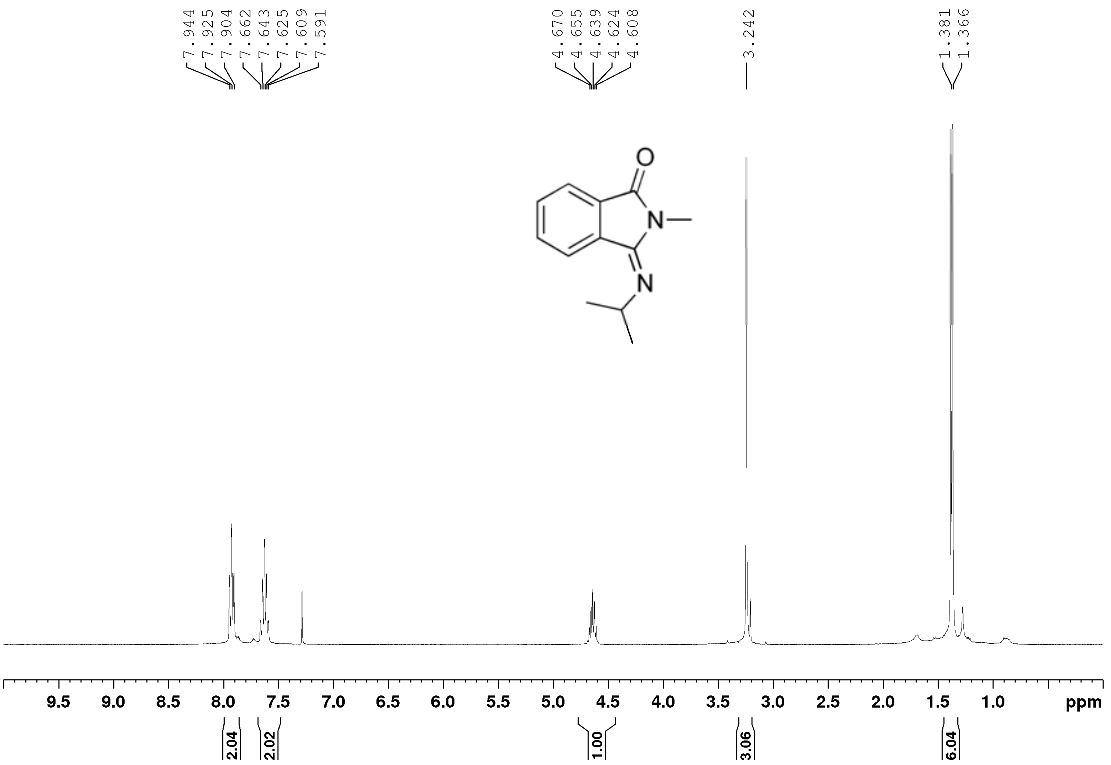

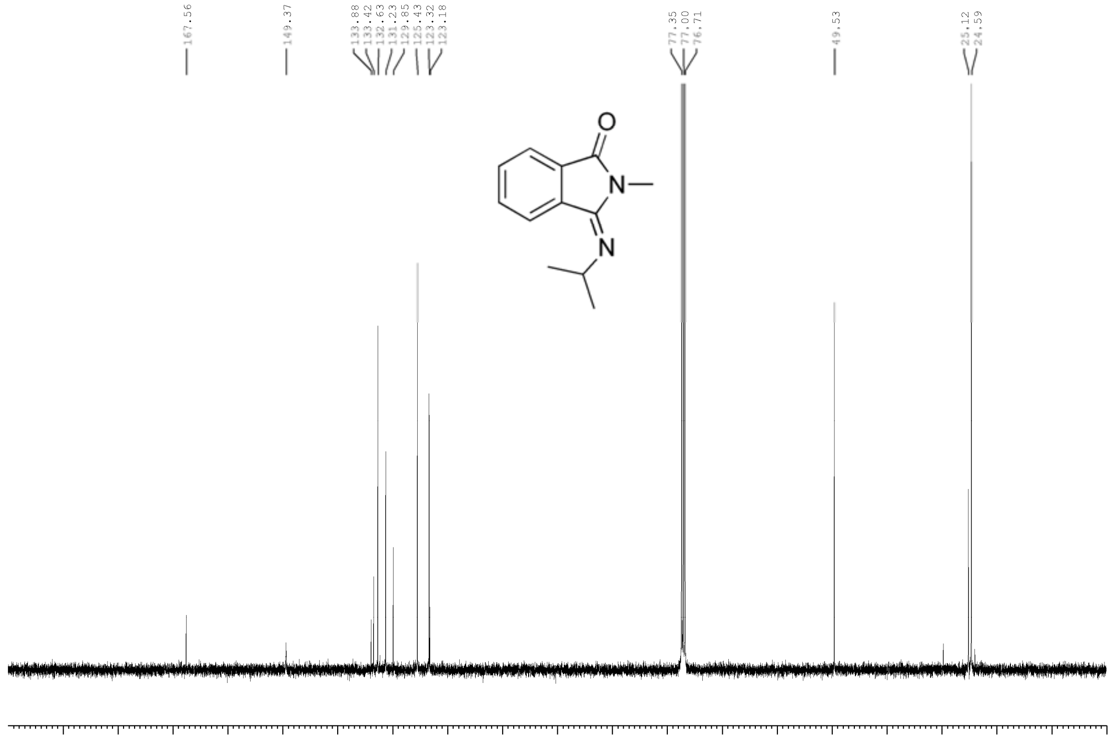

(*E*)-2-(Furan-2-ylmethyl)-3-(isopropylimino)isoindolin-1-one (3o)

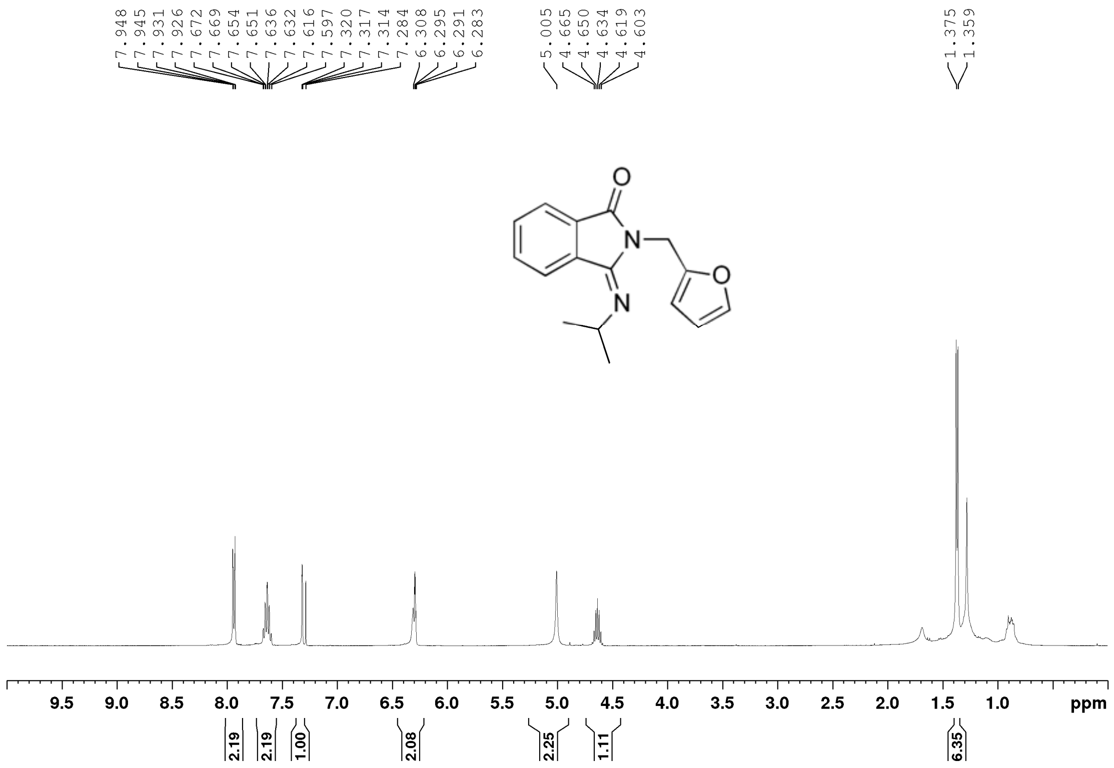

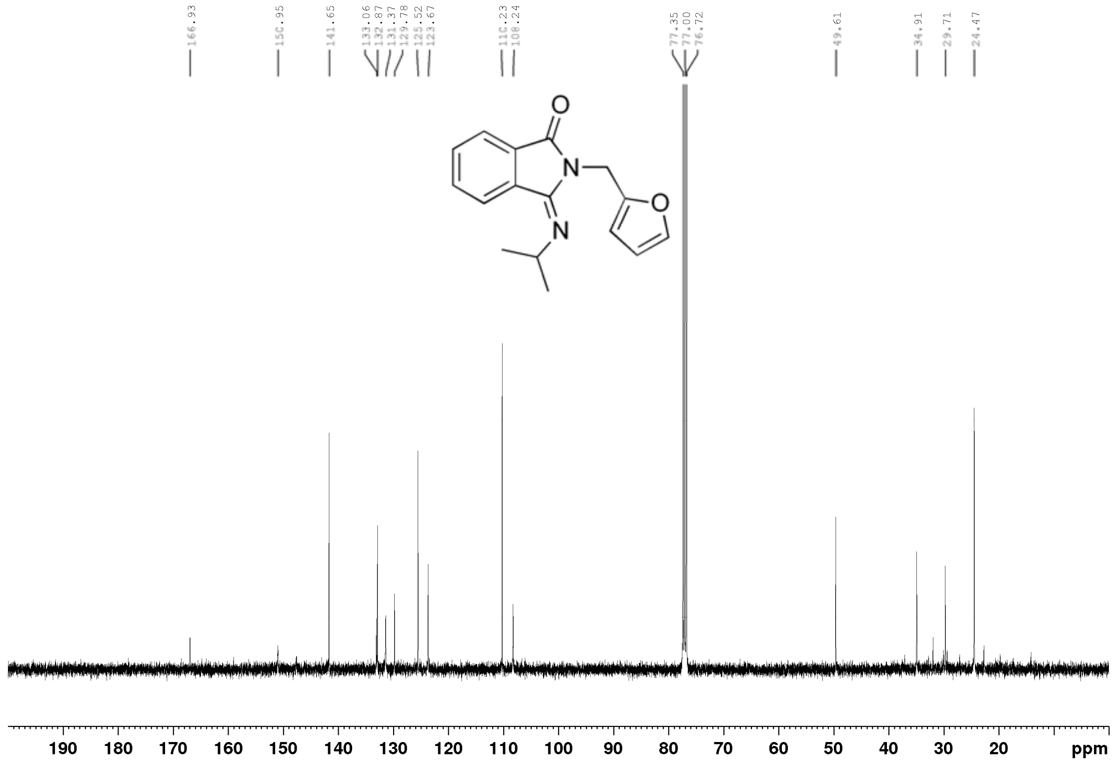

(*E*)-3-(Isopropylimino)-2-(1-phenylethyl)isoindolin-1-one (3p)

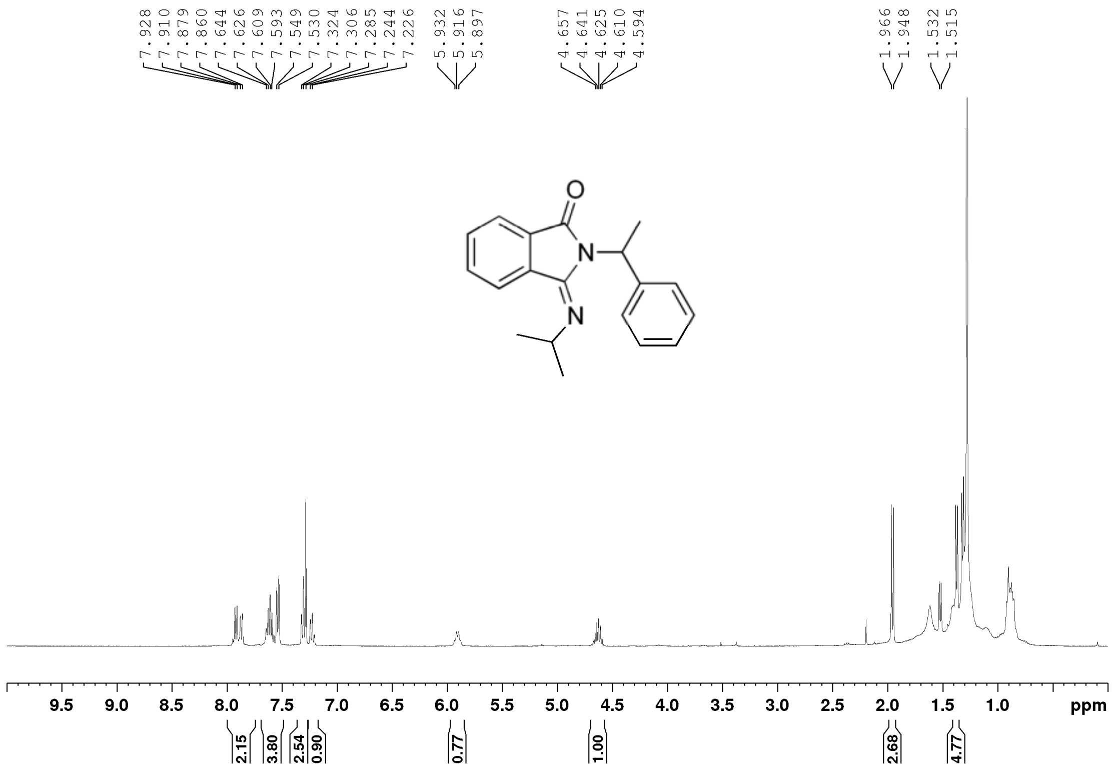

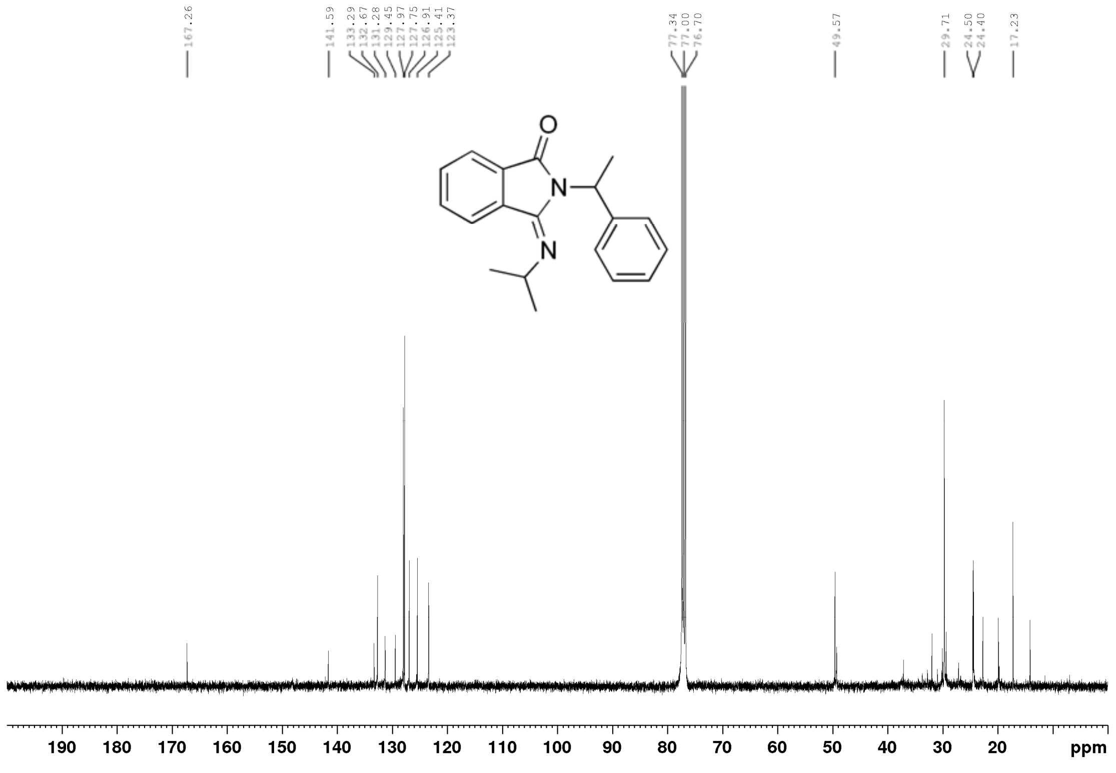

(*E*)-2-Benzyl-3-(isopropylimino)-6-methoxyisoindolin-1-one (3q)

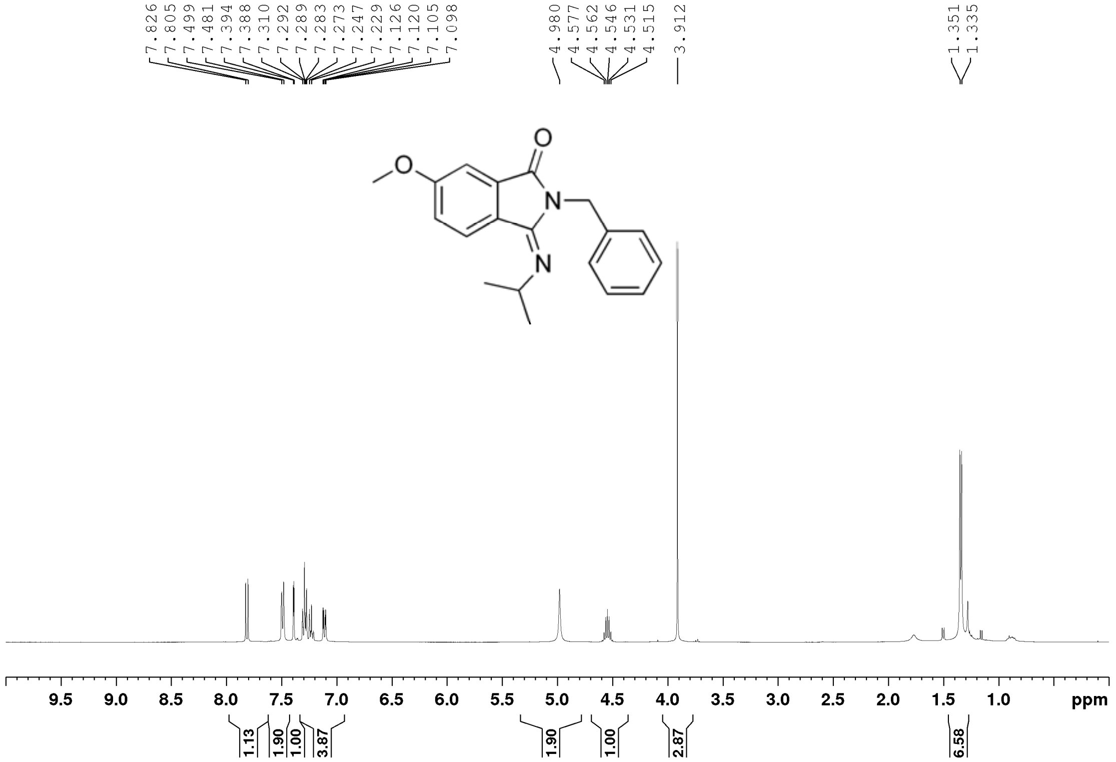

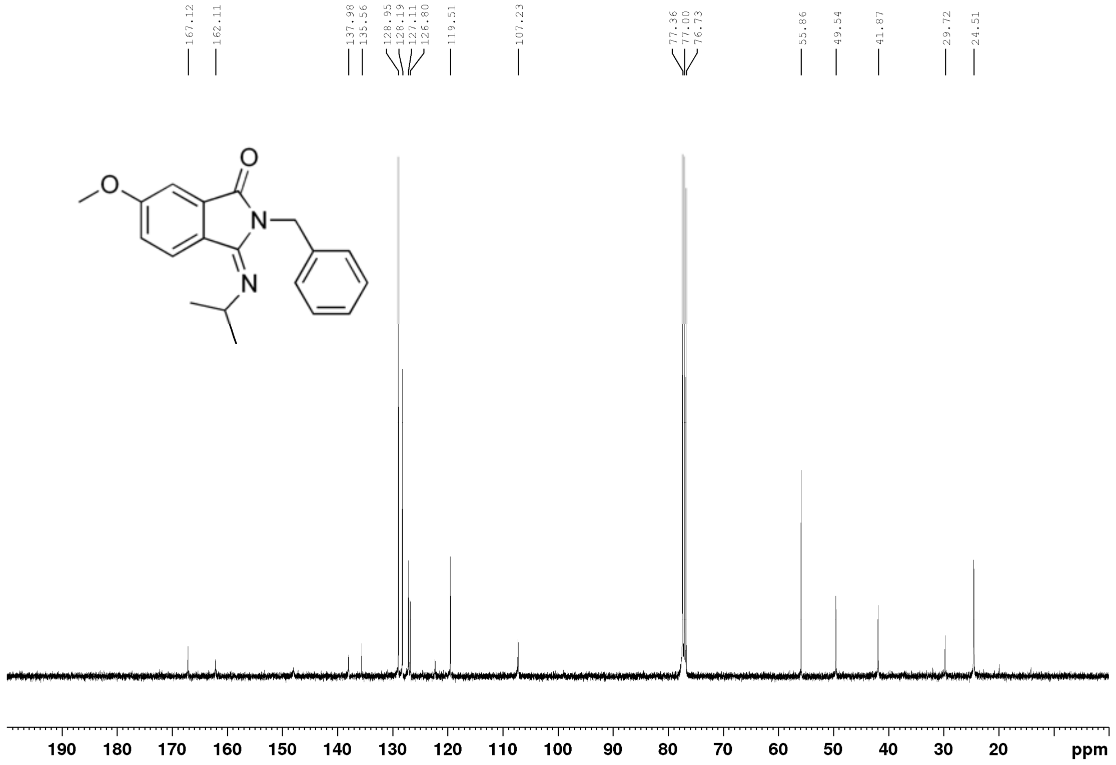

(*E*)-2-Benzyl-5-chloro-3-(isopropylimino)isoindolin-1-one (3r)

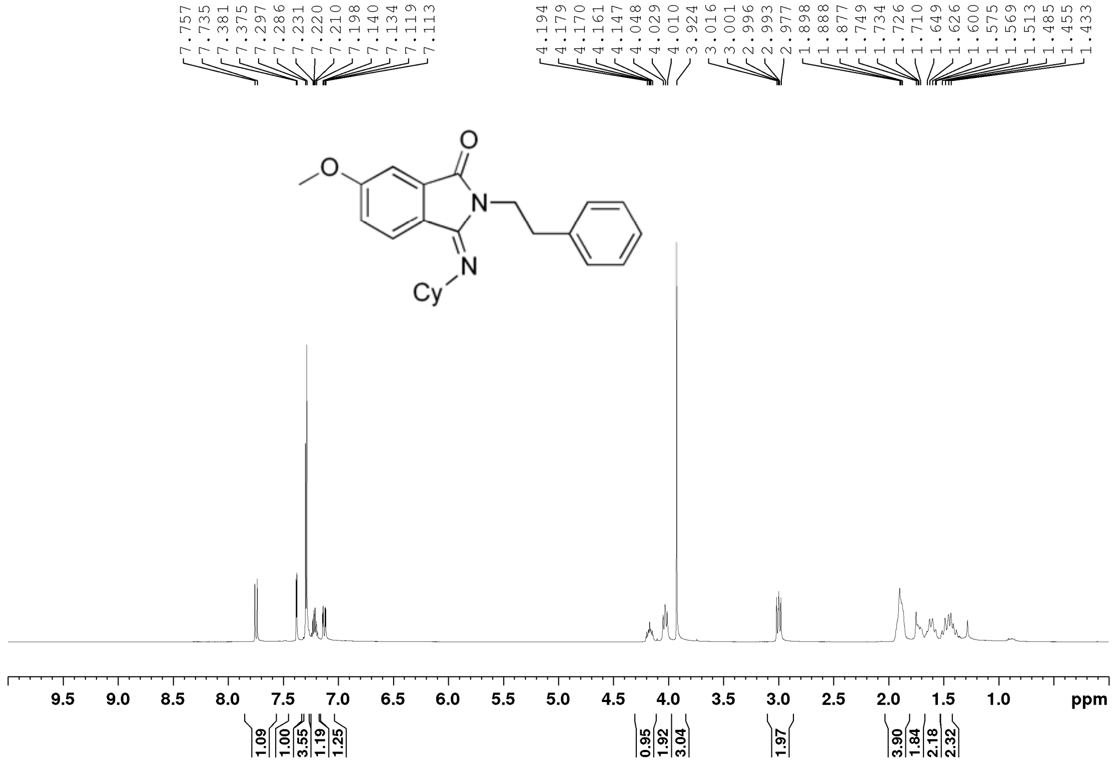

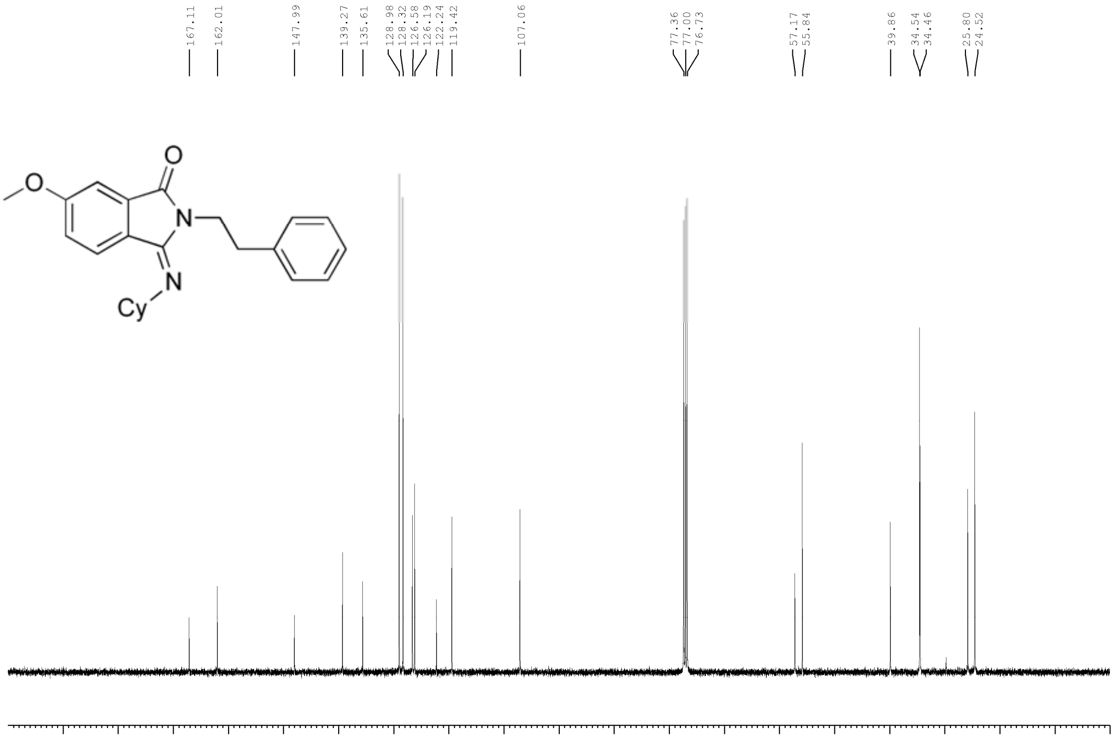

(*E*)-3-(Cyclohexylimino)-6-methoxy-2-phenethylisoindolin-1-one (3s)

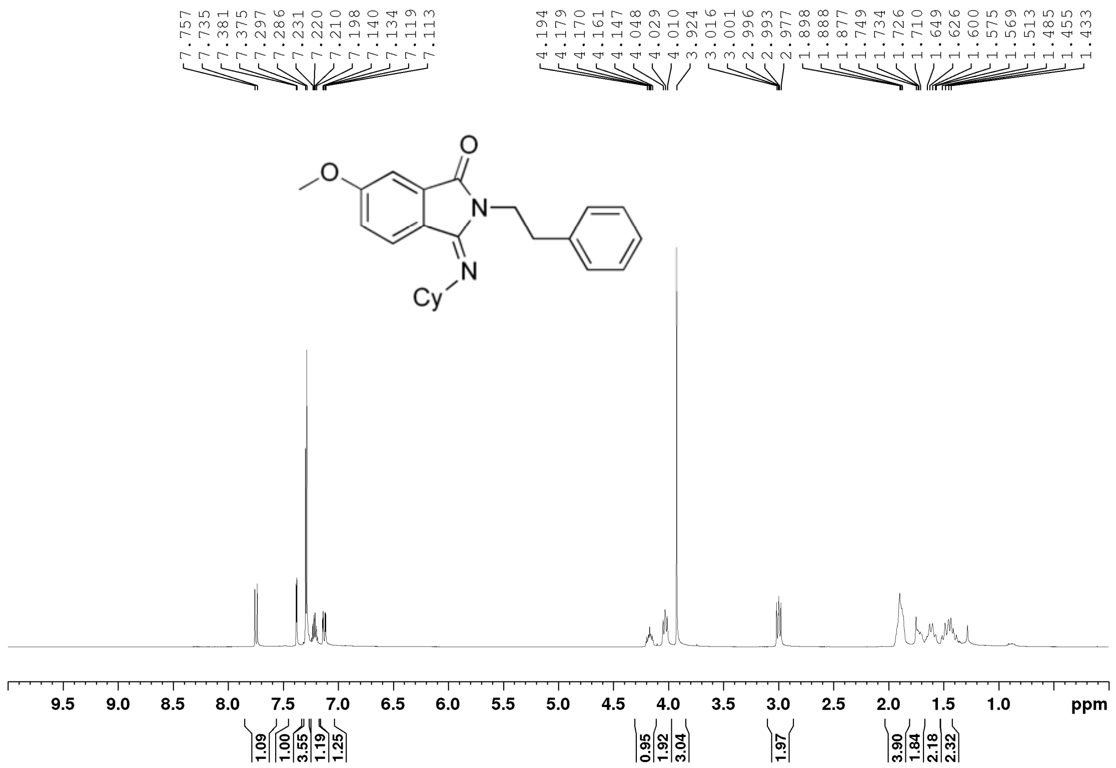

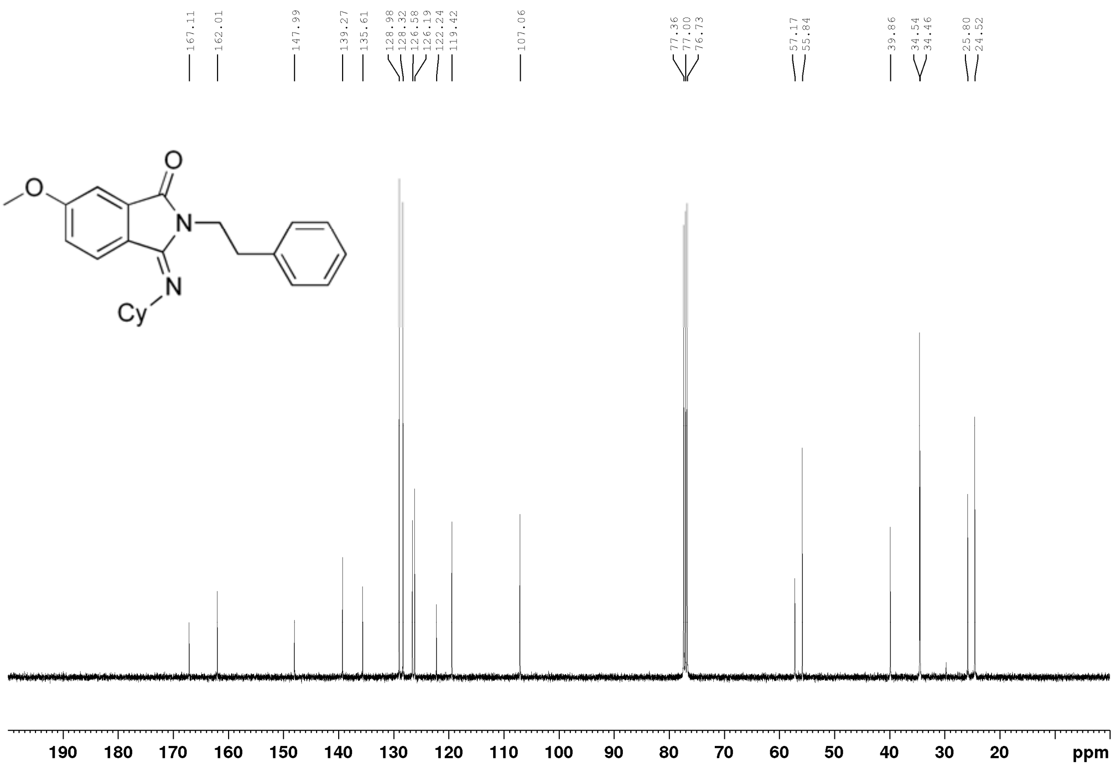

(*E*)-2-Benzyl-3-(cyclohexylimino)-6-methoxyisoindolin-1-one (3t)

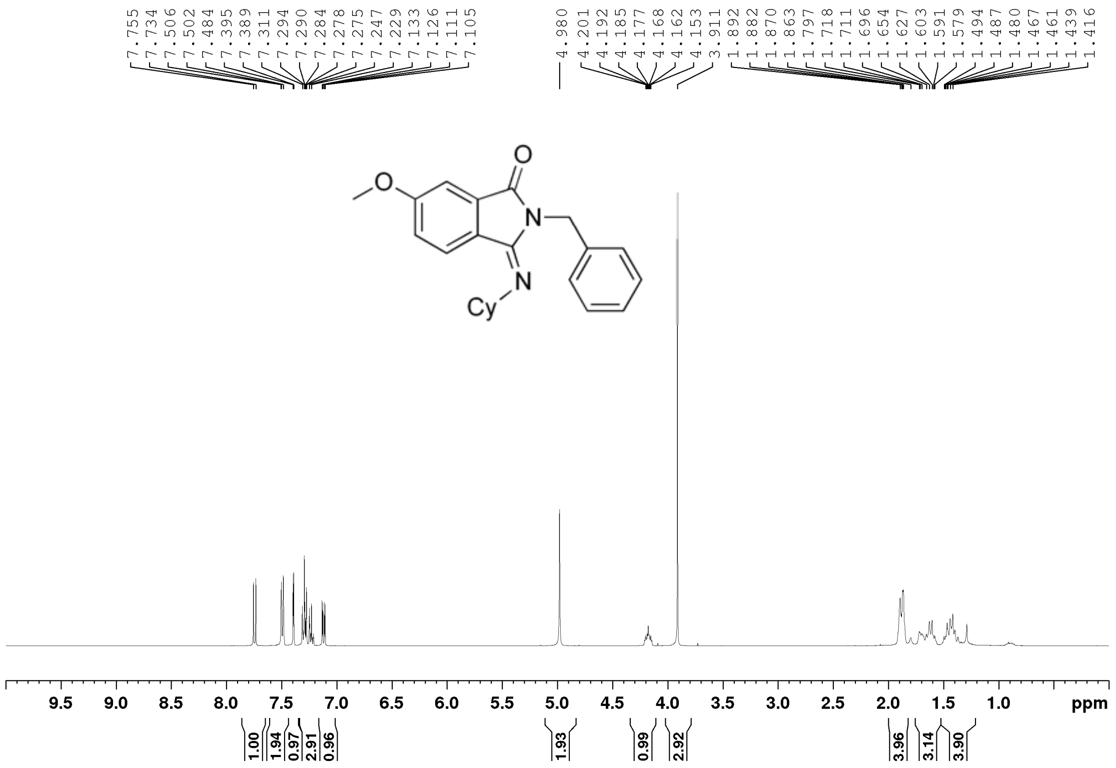

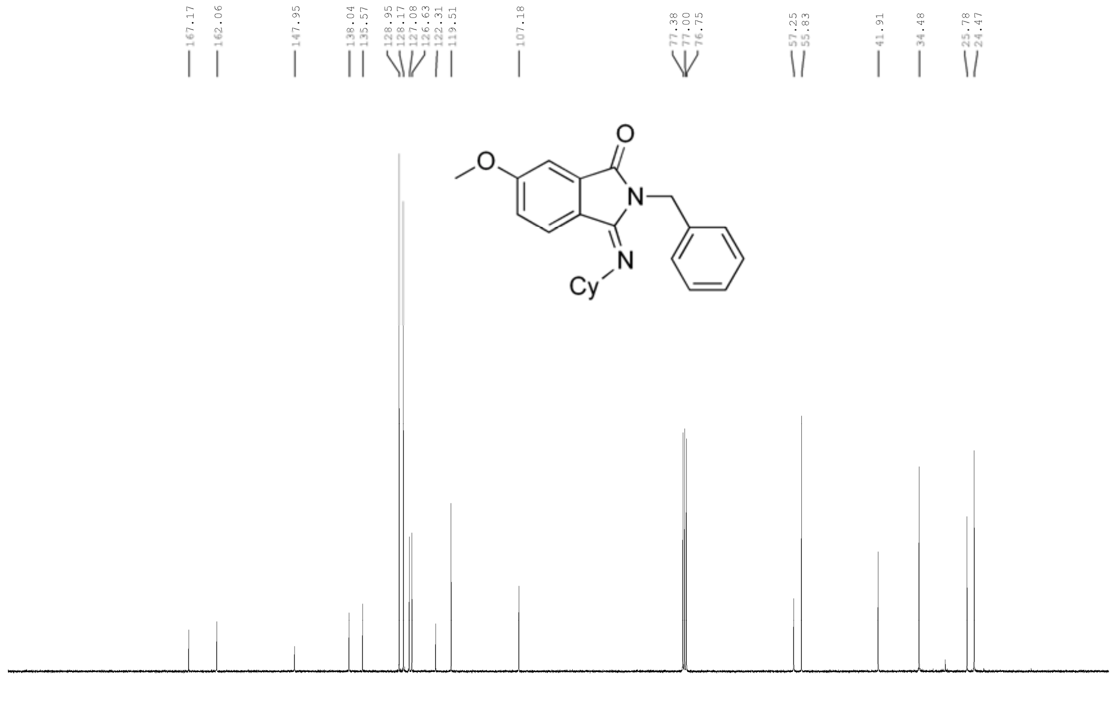

(*E*)-2-(4-Chlorobenzyl)-3-(cyclohexylimino)-6-methoxyisoindolin-1-one (3u)

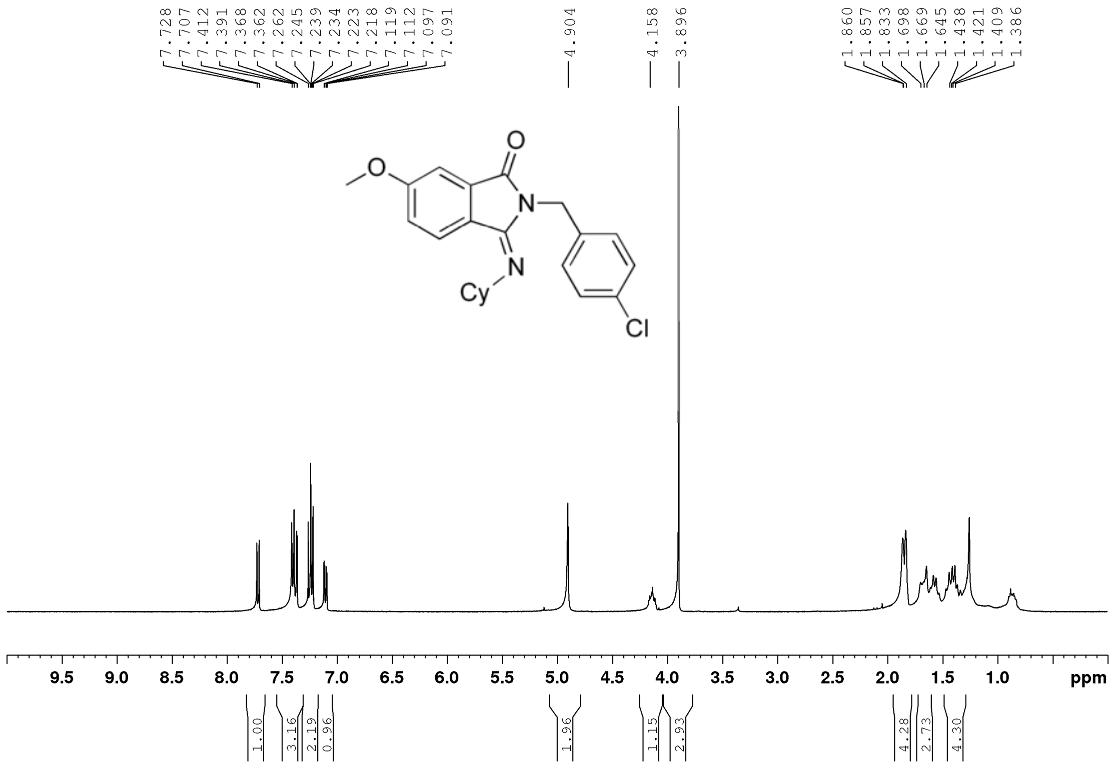

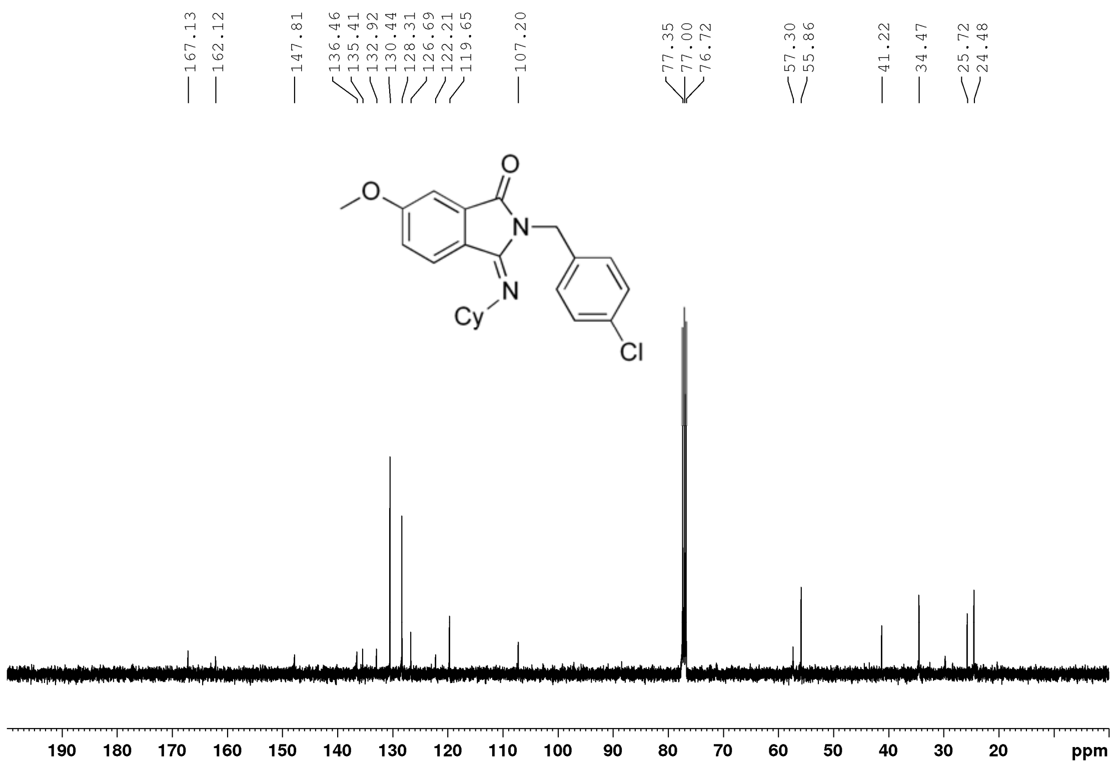

(*E*)-3-(cyclohexylimino)-2-(4-fluorophenyl)-6-methoxyisoindolin-1-one (3v)

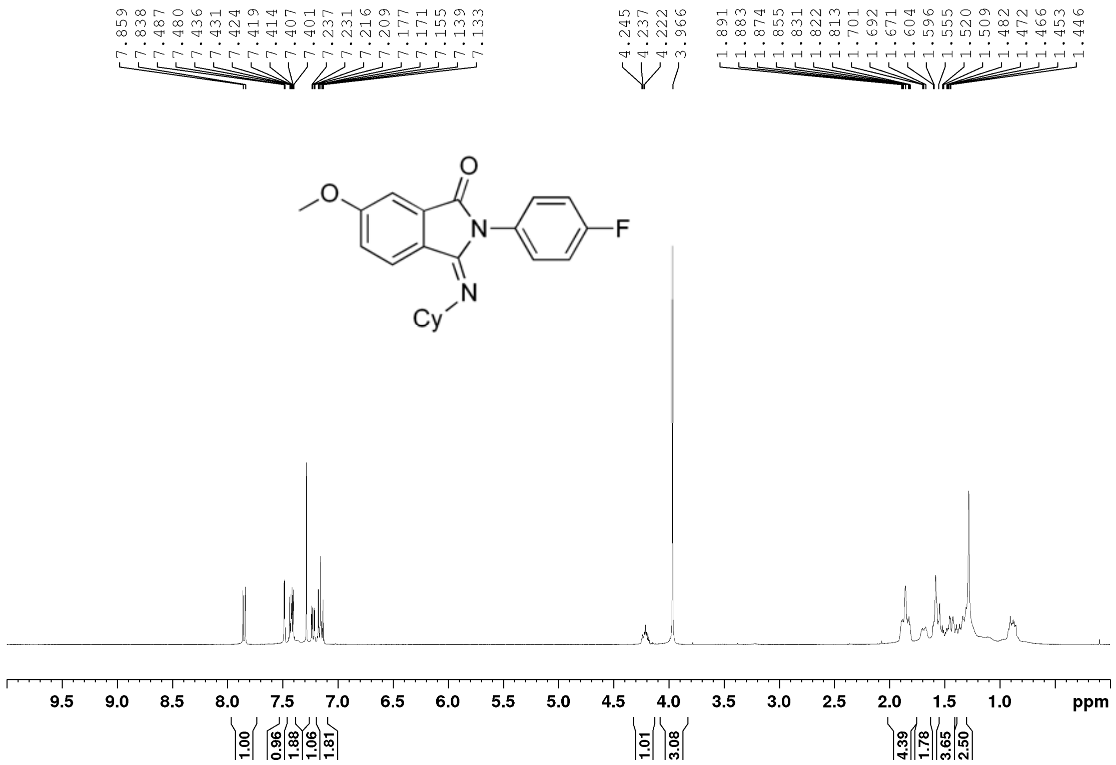

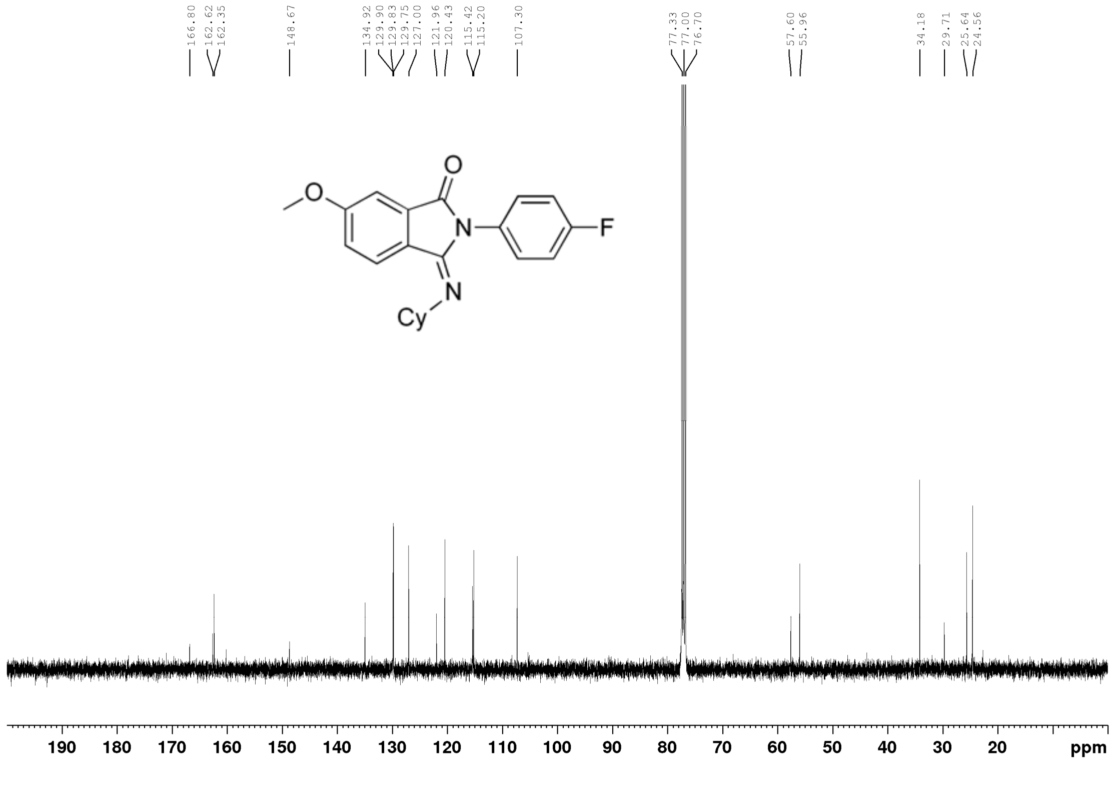

4-Chloro-*N*-methylbenzamide (4j)

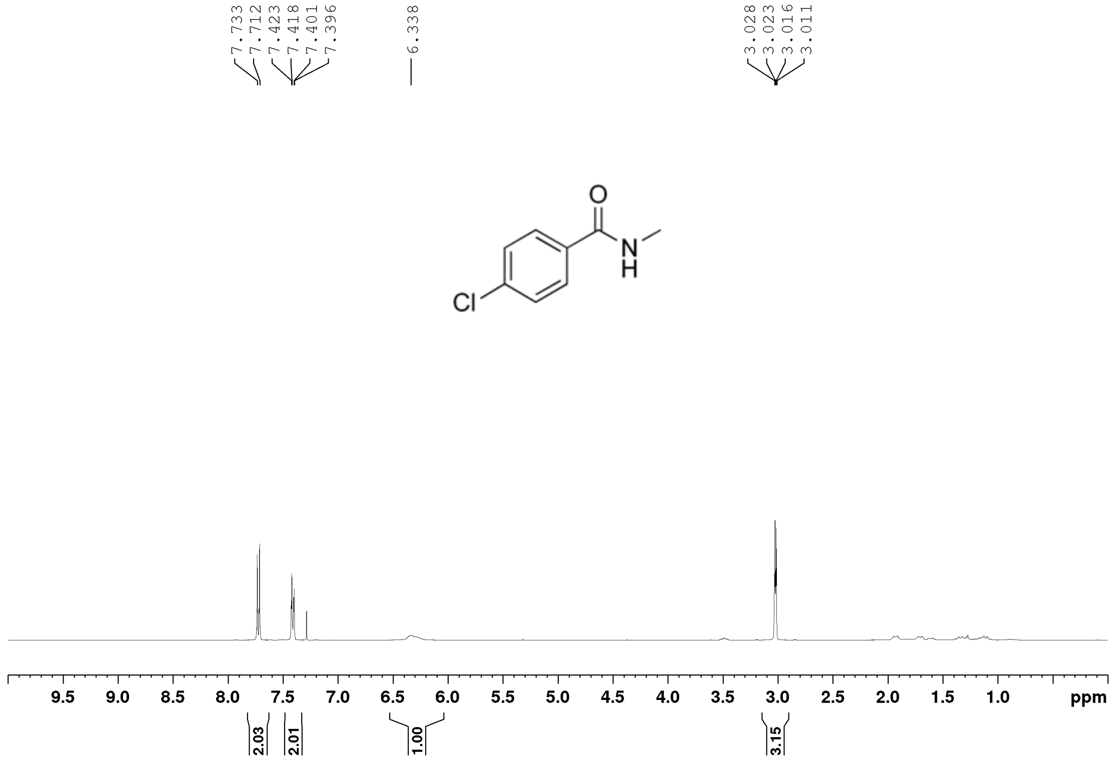

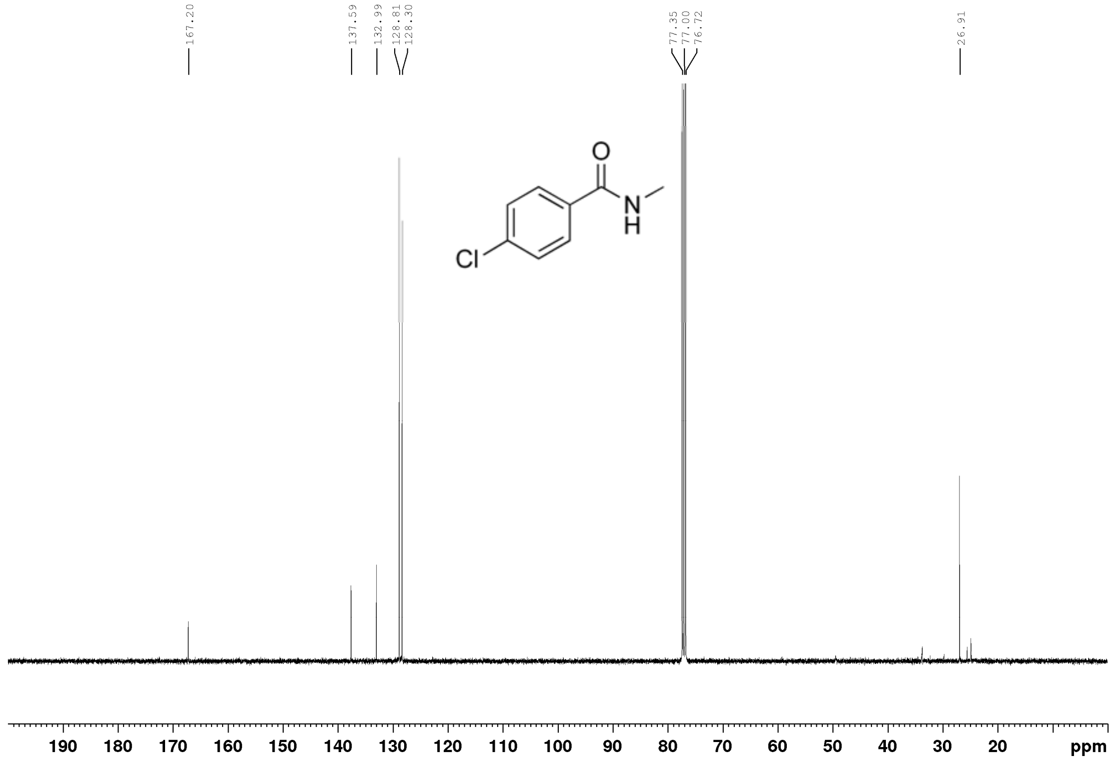

***N*-Methyl-3-(trifluoromethyl)benzamide (4k)**

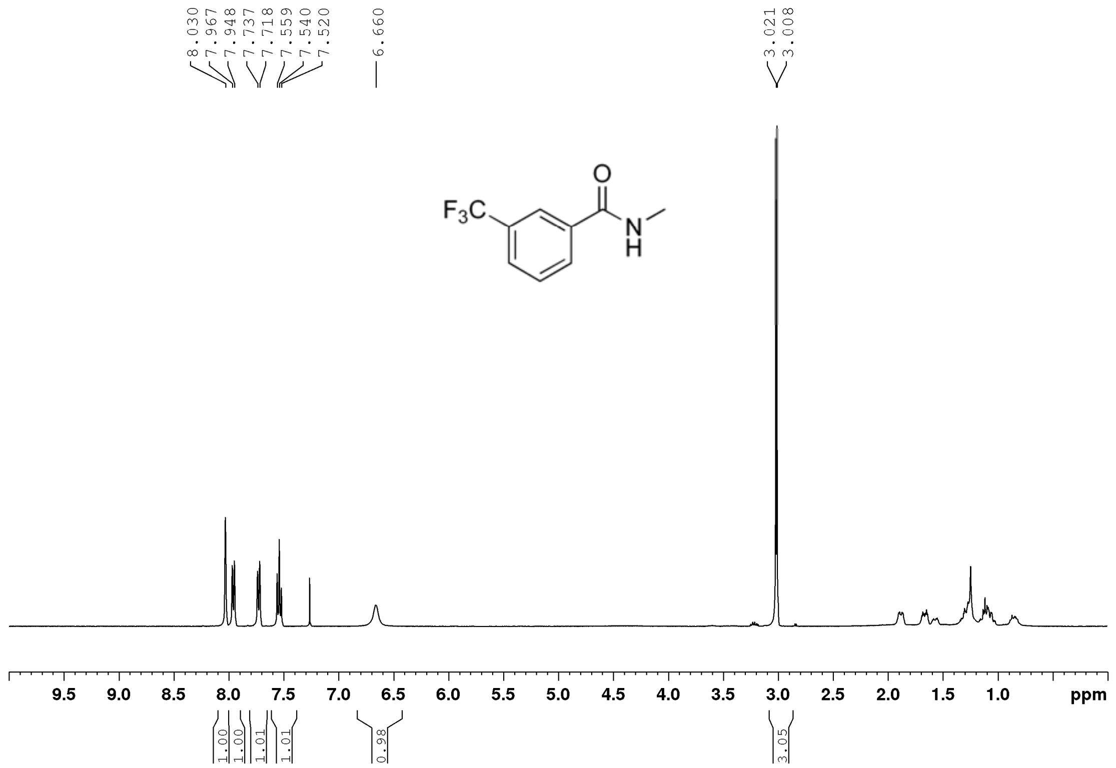

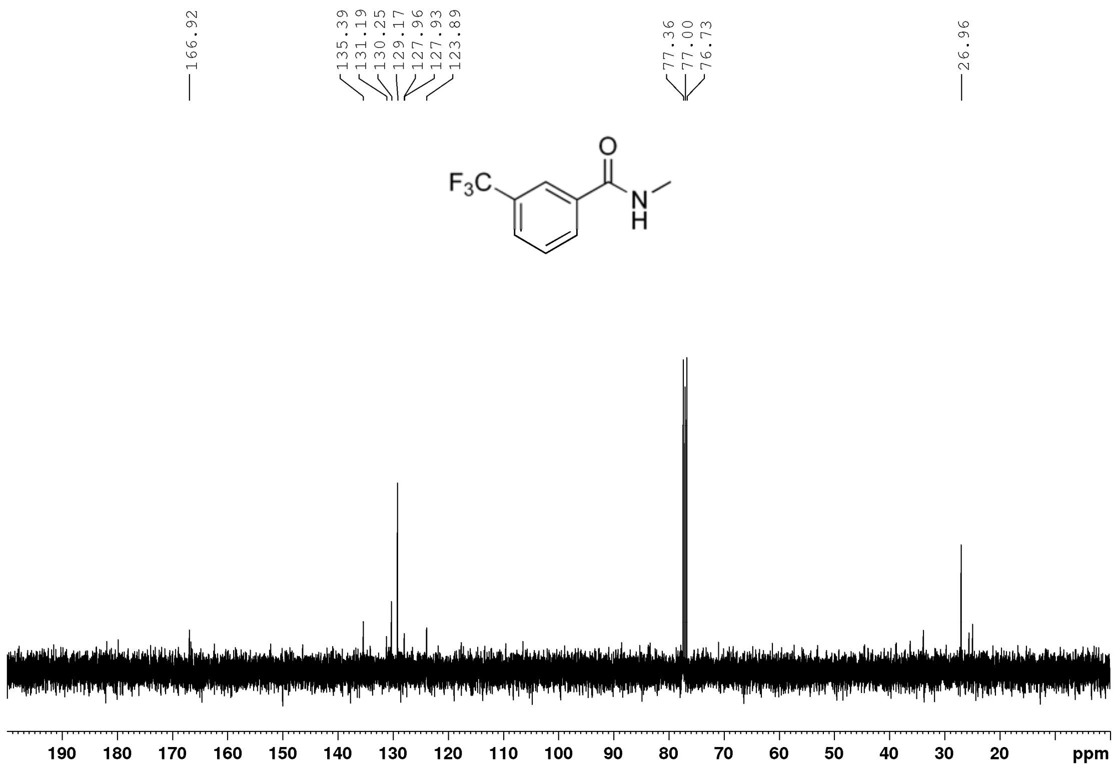

Supplement: Supplementary file 1 [file molecules-26-07212-s001.zip › molecules-1477510-supplementary.pdf]
